# Towards practical radiopharmaceutical treatment planning: a review of dosimetry simplification techniques

**DOI:** 10.7150/thno.132538

**Published:** 2026-08-24

**Authors:** Elmira Yazdani, Zahra Mansouri, Yazdan Salimi, Habib Zaidi

**Affiliations:** 1 Department of Medical Physics, School of Medicine, Iran University of Medical Sciences, Tehran, Iran.; 2 Division of Nuclear Medicine and Molecular Imaging, Geneva University Hospital, Geneva, Switzerland.; 3 Department of Nuclear Medicine and Molecular Imaging, University of Groningen, University Medical Center Groningen, Groningen, Netherlands.; 4 Department of Nuclear Medicine, University of Southern Denmark, Odense, Denmark.; 5 University Research and Innovation Center, Óbuda University, Budapest, Hungary.

**Keywords:** radiopharmaceutical therapy (RPT), theranostics/radiotheranostics, patient-specific dosimetry, single-time-point imaging, digital twins (DTs), physiologically-based pharmacokinetic (PBPK) models, nonlinear mixed-effect (NLME) modeling

## Abstract

Radiopharmaceutical therapy (RPT) plays an important role in modern precision oncology. However, treatment activity is still prescribed by fixed-dose protocols rather than adjusted to planned patient-specific absorbed dose (AD). Individualized dosimetry holds promise for improving treatment planning but is not yet standard. One main reason is that present dosimetry workflows depend on repeated imaging at multiple time points and require kinetic modeling and computational modeling. This review covers recent approaches to make dosimetry more practical and easier to use in everyday RPT workflows.

We introduce a theoretical framework in which simplification methods, which help ease the clinical burden of dosimetry, are classified into three related categories. The first two categories simplify the process through direct reduction in the burdens of either the acquisition or processing of data: (1) Methodological simplifications through reduction in the imaging burden, such as reduced- or single-time point (STP) imaging; and (2) Computational automation via artificial intelligence (AI) to automate dosimetry, including image processing, segmentation, registration, and AD estimation. Advanced modeling strategies in the third category improve efficiency through inference, thereby enabling estimation of AD from sparsely sampled data. Reduced imaging played a major role in defining these areas.

While all of these have their promise, every category of methods has certain drawbacks. STP techniques are less reliable for heterogeneous tumors, black box models have poor transferability and explainability, and digital twin/physiologically-based pharmacokinetic (DT/PBPK) models lack clinical validation. Fundamental dosimetry, with appropriate validity conditions, could allow for reduced reliance on imaging skills and expertise while still retaining sufficient precision for its intended purposes. Standardization of imaging and multicenter analysis is essential for wider adoption of RPTs. A detailed description of models and their effectiveness would be helpful in the clinical application of dosimetry.

## 1. Introduction

Integration of molecular imaging with tailor-made treatments has made radiopharmaceutical therapy (RPT) an essential component of precision medicine 1. Internal radiotherapy involving the use of radionuclides is categorized into systemic RPT, such as peptide-receptor radionuclide treatment (PRRT), including [^177^Lu]Lu-DOTATATE in neuroendocrine tumors (NET), and radioligand therapy (RLT), involving [^177^Lu]Lu-PSMA-617 in metastatic castration-resistant prostate cancer (mCRPC), and locoregional radioembolization, such as Selective Internal Radiation Therapy (SIRT) or Transarterial Radioembolization (TARE). Systemic RPT refers to a process that targets specific receptors/antigens within tumors. In contrast, locoregional radioembolization involves tumor vascularity rather than specific molecular targeting and is performed using radioactive microspheres via a catheter-based intra-arterial method. These procedures may have distinct mechanisms; however, their common denominator is the use of radionuclides for therapeutic purposes 2.

Fixed dosing regimens cannot provide information about the individual differences between patients with respect pharmacokinetic parameters, the biology of the tumor, and the functioning of organs. In particular, absorbed doses (ADs) can vary significantly from patient to patient, which leads to underdosing the tumor or overdosing organs at risk (OARs) 3, 4. Therefore, conventional fixed activity delivery protocols can be ineffective or toxic to patients 5.

An individualized approach based on patient-specific dosimetry enables to overcome the above issues, as ADs are calculated using data obtained via imaging methods and time-activity curves (TACs). Hence, it becomes possible to develop effective individualized treatment protocols 6. However, despite the obvious benefits of internal dosimetry, its application in clinical practice remains problematic due to practical issues related to multi-time-point (MTP) imaging, extensive calculations, and required equipment 2, 7. All this makes even more evident the necessity of further research on reliable methods that would facilitate the implementation of dosimetry in personalized therapy of RPT 8.

One such solution is based on reduced-sampling schemes, including single-time-point (STP) imaging as part of it, where pharmacokinetic models are used for the estimation of the ADs using fewer image acquisitions compared to traditional MTP protocols 7, 8. At the same time, advancements in AI technologies, such as machine learning (ML) and deep learning (DL), help improving the workflow by optimizing certain steps, namely image acquisition and correction through the use of AI algorithms, automatic segmentation and registration without human involvement and with no variability between operators, and AD estimation through DL in place of lengthy Monte Carlo (MC) calculations 9, 10. Apart from AI, alternative advanced computational methods like digital twins (DTs), radiomics, and dosiomics are also being researched 11-14. Though these methods complicate the workflow computationally and theoretically, there is scope for simplifying the workflow through specific inference processes. Inference processes like prediction of ADs from pre-therapy imaging using radiomic and dosiomic models can allow the elimination of post-therapy images from the process. Likewise, by using DT modeling algorithms, it is possible to use less imaging time and arrive at the desired results through inferences made from sparsely available patient data.

Despite the availability of some recent reviews devoted to individual optimization of dosimetric calculations, focusing on simplification of imaging 8, use of AI for automation of the imaging process 15, and using physiologically-based pharmacokinetic (PBPK) modelling for AD calculation 16, to the best of our knowledge, there is lack of comprehensive reviews combining all aspects linked to dosimetry. This review aims to synthesize all these approaches; make critical comparisons, assess advantages and disadvantages of each of them; and discuss the feasibility of applying each approach in practice in dosimetry.

In other words, the goal of this review is to fill the research gap and conduct a narrative analysis of existing methodologies that are employed to improve the process of performing internal dosimetry from a clinical point of view. Particular focus will be on reducing the number of imaging time points, automating manual intervention, and fast calculation of ADs. Yet, the scope of this review does not cover just one methodological aspect. We will consider the following methods, namely STP dosimetry, AI-automated approaches, PBPK models, and DT-based approaches under one concept.

By highlighting the relationship between these areas and focusing on their integration, this review hopes to go beyond the piecemeal approach to offer an outline for more efficient dosimetry techniques in RPT.

## 2. Radiopharmaceutical Therapy and Dosimetric Considerations

The limitations of non-individualized therapeutic procedures have led to the need for more dependable patient-specific RPTs 17, 18. The dosimetric approach is capable of providing an accurate estimation of ADs delivered to the tumor and OARs by utilizing images, drug pharmacokinetics, and patient anatomy. Two major steps characterize a dosimetric approach: (i) characterization of the radiation source, i.e., the distribution of radioactivity in the patient's body, and (ii) the modeling of radiation transport from the radiation source to energy absorption in the target 19. It is pointed to highlight the separation between these two problems to discuss strategies for simplification discussed in Section 4. Source characterization can be simplified in terms of the reduction of image acquisition, population-based, or PBPK models, as well as the usage of prior cycle scaling. Radiation transport modeling could be simplified by automated AD calculation through DL, kernel methods, or accelerated MC techniques using GPUs 20 or neural networks 21. Some methods, such as AI-based voxel dosimetry and DT models, are able to solve both tasks simultaneously.

There has been remarkable development in the field of RPTs within the past ten years 2. Various radionuclides that emit β⁻ radiation have been employed for labeling treatment agents, such as [^177^Lu]Lu-PSMA-617 RLT, which is used in the treatment of mCRPC, and [^177^Lu]Lu-DOTATATE PRRT that is US Food & Drug Administration/ European Medicines Agency (FDA/EMA)-approved for the management of NET tumors expressing somatostatin receptors (SSTR) 19, 22. Moreover, many other labeled radiopharmaceuticals have already obtained approval (see Table 1), while others have either pending FDA/EMA approval or are undergoing an expedited evaluation process 15, 23-28.

Dosimetry-related issues raised in Table 1 will be considered in Sections 3 and 4. In Section 3, the conventional process employed by all types of RPT is described. In Section 4, methods that can be used to overcome these difficulties include image reduction, AI, and more advanced models, and some radiopharmaceutical applications highlighted from the literature.

**Figure 1:**
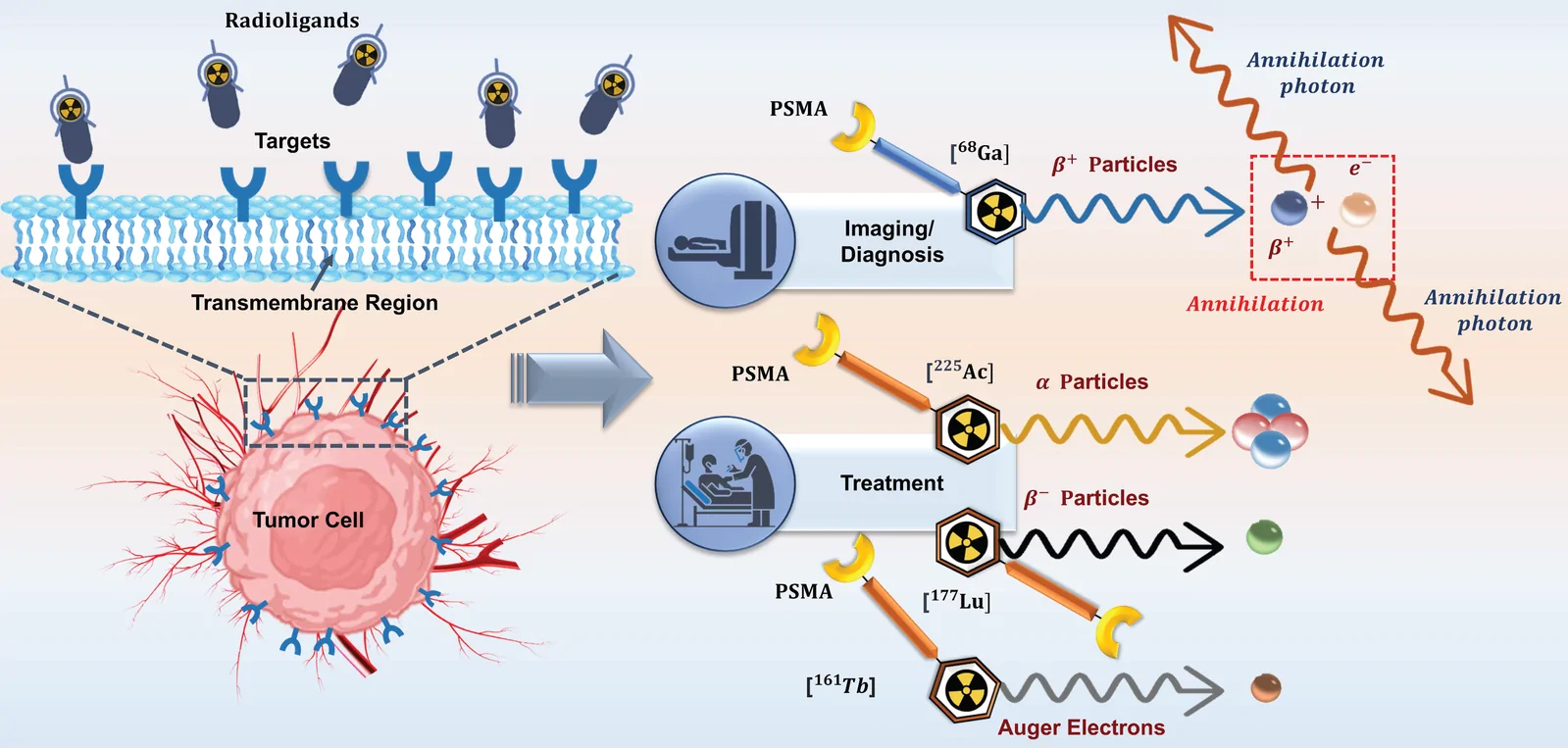
Schematic illustration of radiotheranostics. A single targeting ligand binds tumor-specific receptors and can be labeled with diagnostic ([^68^Ga]Ga-PSMA-11) or therapeutic ([^177^Lu]Lu-PSMA, β^-^-emitter; [^225^Ac]Ac-PSMA (α-emitter); [^161^Tb]Tb-PSMA (Auger electron emitter) radionuclides, allowing integrated imaging and treatment of prostate cancer.

Figure 1 shows an example of a radiotheranostic model, where a single targeting moiety is labeled with imaging and therapeutic radionuclides, and the same molecule can detect and treat tumors expressing the corresponding receptors. Diagnostic nuclides, including ^68^Ga, typically release positrons that collide with electrons to produce a pair of annihilation photons with an energy of 511 keV. One specific compound, called the PSMA ligand, is considered a theranostic pair since [^68^Ga]Ga-PSMA can be used for diagnostics via positron emission tomography (PET) while [^177^Lu]Lu-PSMA is employed for therapy via RPT in patients with mCRPC.

Predictive modeling of the therapeutic biodistribution and ADs using pre-therapeutic quantitative PET images has also been widely applied recently 49-54. However, this method should be used with caution because diagnostic and therapeutic agents can vary in their peptide size, ligand, and kinetics; the scaling assumption may require verification; and the changes in biology from one scan to another can also influence biodistribution. Hence, predictive models need to be verified for each theranostic pair.

The therapeutic benefit of alpha and beta-emitting radionuclides, such as ^177^Lu and ^225^Ac, is attributed to their higher accumulation in the lesion site. On the other hand, there is comparatively less uptake of radionuclides by healthy tissues, thereby pointing out the need for dosimetry. In the case of RPT, quantitative serial imaging can lead to accurate estimation of ADs and provide personalized activity estimation along with organ-sparing plans. The concept of dosimetry can be applied in multi-cycle therapy regimens, where dose calculation is possible during the treatment process, usually either during the first cycle or in other cycles after the first one, to improve AD estimation 5, 55. Nevertheless, performing only one cycle means assuming the distribution pattern found in the first cycle is reflective of all others, an assumption that can cause large margins of error 19, 22.

The treatment of both primary and metastatic hepatic malignancies with radioembolization is commonly done with the use of [^90^Y]Y-labeled resin or glass microspheres. The treatment plan generally requires pre-procedural simulation using [^99m^Tc]Tc-macroaggregated albumin (MAA) or in some cases, even with the therapeutic microspheres, and later confirmation of microsphere distribution post-procedure with quantitative imaging 56. This helps in ensuring optimal balance between tumor radiation dose and protection of the normal liver and lungs 15, 57.

α-radiopharmaceuticals have recently been of great interest, especially [²²⁵Ac]Ac-PSMA for prostate cancer 58, 59, [²²⁵Ac]Ac-ABD-147 for lung and NETs, and [²¹²Pb]Pb-VMT01 for metastatic melanoma 15. The effectiveness of α-emitters is based on their greater linear energy transfer (LET) and higher stopping power, allowing for a more pronounced effect on tumors with minimal injury to the surrounding healthy tissues. Due to their small range and high LET, α-emitter radiation is known to create a considerable degree of inter- and intra-voxel AD variability, demanding more complex treatment strategies for effective tumor control and maintaining low doses of radiation exposure for healthy tissues 15.

Radiopharmaceuticals using radium-223 (^223^Ra), including [^223^Ra]RaCl2 (Xofigo), is used for targeting bone metastases that develop secondary to breast or prostate cancers. The dosimetry of ^223^Ra has always been limited because of some of the attributes associated with this alpha-emitting agent 15. Although gamma rays only account for 1% of the total emitted energy by ^223^Ra, there seems to be growing evidence supporting the use of ^223^Ra quantitative imaging 60.

Furthermore, [^161^Tb]Tb-PSMA, which emits β⁻ along with Auger electrons (AEs), deposits energy at the sub-cellular level and therefore has proven to be highly effective for treating mCRPC 47. In such cases, wherein the bone marrow remains as the AD-limiting organ, highly specialized dosimetric methodologies are required. These dosimetry approaches should take into account micro- and sub-volume energy deposition.

Nevertheless, dosimetry in RPT faces challenges due to the time-varying biodistribution of the radiopharmaceutical. Such biodistribution requires complex mathematical modeling and lengthy procedures for accurately computing the AD. It is therefore vital to develop efficient approaches that allow widespread use of personalized dosimetry in RPT, where standard methods involving MTP imaging with manual and computationally demanding procedures have not succeeded in addressing these limitations.

## 3. Traditional Internal Dosimetry Workflow: Challenges and Limitations

**Figure 2:**
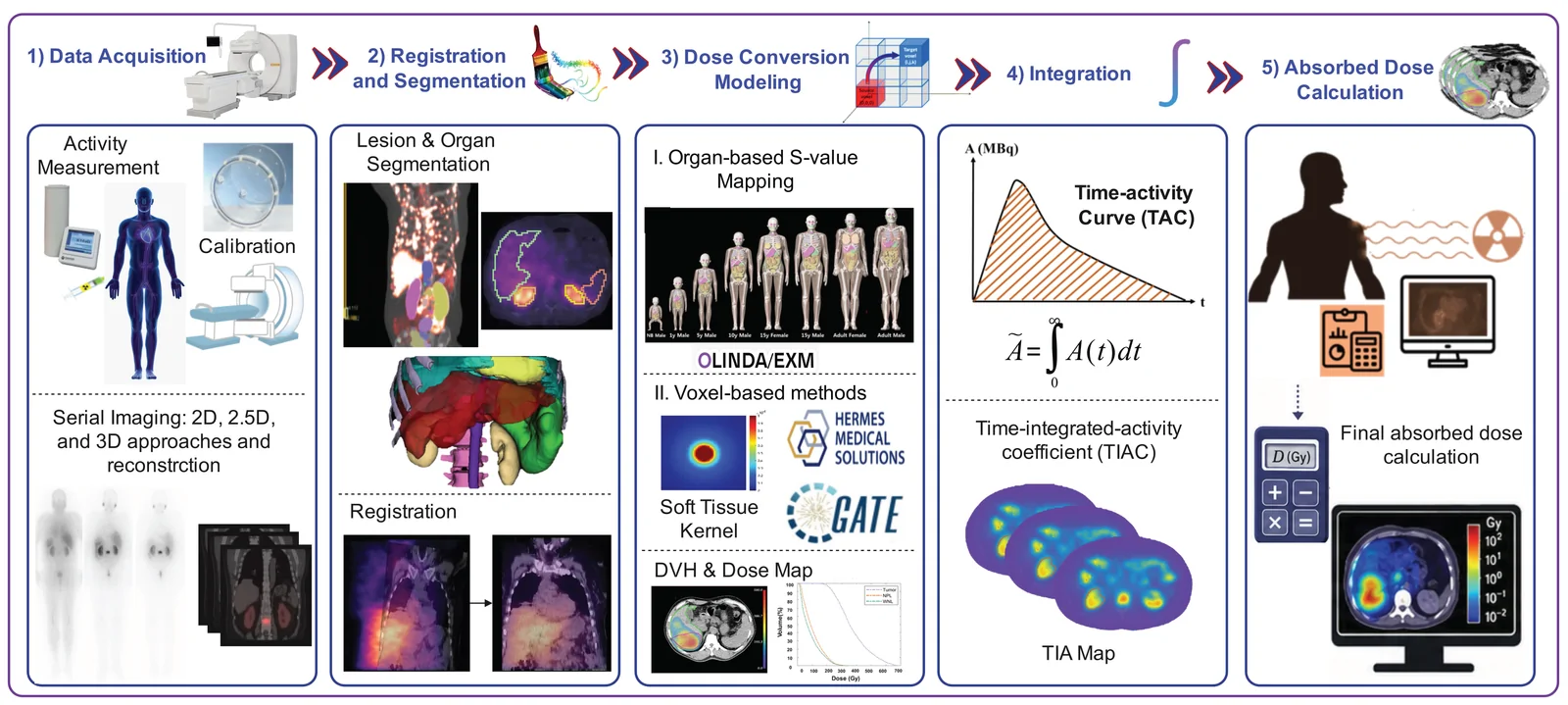
Overview of the five key steps in the patient-specific dosimetry workflow for radiopharmaceutical therapies: data acquisition, segmentation and registration, dose-conversion modeling, integration, and absorbed dose calculation. Note that the sequence of steps is method-dependent and not necessarily in this order.

RPT makes it possible to make informed decisions regarding the activity dose delivery since it considers variations among patients as well as intra-patient variations with the aim of optimizing the dose delivered to tumors with minimum impact on OARs 1, 47. However, there are numerous clinical and technical challenges faced within the conventional treatment framework that prevent its regular implementation. Personalization of treatment based on dosimetry can be divided into five critical phases, each of which has its own limitations, thus motivating the introduction of simplifications described in section 4 61. These stages include data acquisition, registration and segmentation, modeling of AD conversion, integration, and finally AD calculation. This can be visualized using the scheme depicted in Fig. 2 below. It should be noted that the order of steps may differ depending on the technique being used.

### 3.1. Data Acquisition

To calculate the net administered activity of radiopharmaceuticals, it is necessary to measure their activities before and after injection using a dose calibrator that has been appropriately calibrated. Calibration of the gamma cameras plays an important role, and the process of data manipulation for image reconstruction should incorporate detector response, dead-time corrections, photon attenuation and scattering, and partial-volume effects (PVE) correction. In sequential quantitative imaging, it becomes possible to study the behavior of radiopharmaceuticals across time as well as the different organs or tissues 62. Based on the scanner available, imaging can be performed through sequential planar whole-body scanning, sequential SPECT/CT, or even a combination of both, known as the 2.5D technique 62.

The general recommendations for optimum imaging time points are presented in the Medical Internal Radiation Dose (MIRD) Pamphlet 16 63. Guidelines on the use of quantitative SPECT for patient-specific dosimetry are outlined in MIRD Pamphlet 23 64, whereas MIRD Pamphlet 26 provides more details about SPECT quantification for ^177^Lu RPT dosimetry 65.

**Limitations:** Traditional MTP protocols involve at least 3-4 scans within 4-7 days, resulting in high costs, reduced compliance, limited availability of scanners, and high workloads for health-care personnel. Even with optimized protocols, the long duration of acquisitions (about 25-30 minutes per bed) is another obstacle that causes patient discomfort and leads to motion artifacts. These constraints can be solved by decreasing SPECT acquisition time (Section 4.1) and decreasing time point acquisitions (Section 4.2).

### 3.2. Registration and Segmentation

The tumors and OARs are outlined to establish volumes of interest (VOIs) that are used for determining the tissue mass and dosimetry. Serial images are coregistered to facilitate propagation of the VOIs and ensure consistent anatomical placement between the different time points. The quantitative data are acquired based on these VOIs, yielding discrete biokinetic values of the selected time points. Then, the TAC can be determined by fitting a continuous function (e.g., sums of exponentials and compartment model) to these discrete biokinetic values. This registration process is essential to facilitate correlation between the correlated voxels during voxel-wise TAC fitting.

**Limitations:** Manual segmentation is labor-intensive (takes 30–60 minutes per patient), suffers from high intra-/inter-reader variability; and demands a specialized skill set. Rigid registration cannot be performed for anatomically deformable areas (abdomen, head/neck). This is the rationale behind automation in both segmentation (Section 4.4) and registration (Section 4.5).

### 3.3. Dose-Conversion Modeling

These models provide information about the conversion of energy released by radiation and absorbed in the target location (AD). The S-value approach is a widely used method for dose conversion modeling, which estimates the energy released by each nuclear reaction in a target region as a function of the mass of the target region and the fraction of energy released by the radioactive source that is absorbed by the target 66. The S-value is mathematically defined as (in Gy/Bq·s or mGy/MBq·s units):


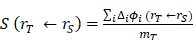
 (1)

where 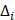 is the mean energy emitted per nuclear transformation for radiation type 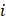, 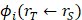 is the fraction of that energy absorbed in the target region 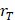 from the source region 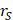, and 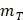 is the mass of the target region.

S-values can be provided in two primary forms. Pre-calculated organ-level S-values tabulated for reference phantoms allow quick AD computations for standard source-target pairs and hence can be used in regular clinical practice if the patient’s anatomy only slightly differs from that of the reference model. On the other hand, voxel-based S-values (VSVs) can be calculated on a voxel-by-voxel basis considering density and composition data obtained through a patient’s CT. VSVs allow calculating more accurate and patient-specific AD values in heterogeneous tissues and near their boundaries when the contribution of cross-AD effects is high 66. VSVs become critical in the case of α-emitting and Auger-electron emitting radionuclides due to their short-range and sensitive local AD to density variation. Several additional methods of dose-conversion modeling, not involving the use of S-values, have been developed.

**Limitations:** The S-values are determined based on standardized phantom anatomic structures that ignore the mass, volume, and heterogeneity of the tissues in the patient’s body, thus resulting in errors, particularly near tissue boundaries and small organs. Although the precomputed values for VSVs provide better accuracy, they require large amounts of computation time. Such limitations can be overcome by using DL to estimate AD (Section 4.6).

### 3.4. Integration

Time-integrated activity (TIA) indicates the sum of all decays from the source region expressed in units of MBq.h (or cumulated activity). The TIA coefficient (TIAC) is determined by dividing the TIA by the initial administered activity as the initial state (A_0_), resulting in a dimensionless value expressed as time (e.g., hours; TIAC (h) = TIA/A_0_. TIAs can be computed using any of the following three approaches:

**(1) Curve fitting with analytic integration (less complex):** Both mono-parametric and bi-parametric exponential curves are used for modeling of the discrete biokinetic data; the TIAC value is then computed using analytical calculation of the fitted curve. This methodology relies on a parametric function describing clearance kinetics 63.

**(2) Numerical integration (intermediate complexity):** Direct application of the trapezoidal rule or Simpson's rule to the biokinetics data by extrapolation beyond the last data point (e.g., assuming physical decay or a mono-exponential tail). Such a method does not require any parametric assumptions about the nature of dependence between the data points, but caution should be exercised in case of late-phase extrapolation 5, 63.

**(3) Compartmental modeling (most complex)**: Ordinary differential equations describe radiopharmaceutical kinetic behavior in an interrelated system of compartments (e.g., blood vessels, organs, and tumors). The parameters are estimated by performing a least-squares fit of the mathematical model to the entire dataset; then TIACs are derived from the solutions of compartments 18, 67. This method allows TIAC estimation of those compartments that were impossible to quantify due to insufficient sensitivity and resolution, overlapping activity, or inadequate sampling rate. These are inferential TIACs according to the structure of physiologically-based models and kinetic constraints on the parameters of the transfer rates. Accordingly, parameter estimation and the structural integrity of the model remain important challenges, particularly in cases with sparse datasets 16, 68, 69. Guidelines on time point selection for imaging and sampling frequency for establishing TACs are provided in MIRD pamphlet No. 16, also referred to as the single S-value (SSV) approach 63.

**Limitations:** The computation of TIA requires complete information about the whole clearance process. Fitting the curve can be affected by time points chosen, interpolation issues, and noise. Moreover, bi-exponential fits can suffer over-fitting issues with insufficient data. The above problem can be overcome using STP methods (Section 4.2). Such techniques include nonlinear mixed-effects (NLME) modeling (Section 4.2.4) and data-driven techniques like GAM/ML (Section 4.2.5).

### 3.5. Absorbed Dose Calculation

The last step in the process is the calculation of the AD to tumors and OARs 70. In general, AD estimation at the organ level is performed according to the MIRD model. Hence, the mean AD for a particular target region can be obtained by combining the TIAC from Step 3.4 with the respective S-values from Step 3.3, as shown in Equation (2).


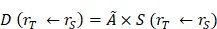
 (2)

Three possible approaches exist for calculating AD from voxelized activity distributions, including local energy deposition (LED), dose-point kernel (DPK) convolution, and full MC radiation transport. Recommendations given in the ICRU Report 96 outline useful guidelines for making this decision 71. If the deposition of energy occurs essentially within the voxel of origin and does not affect other regions through crosstalk effects, LED can be used. However, when radionuclide energy deposition leads to greater penetration and increased photon yield, resulting in significant crosstalk effects in other regions, it becomes necessary to use DPK convolution or MC methods. When the media in which radiation propagates can be considered homogeneous, then DPK convolution is appropriate, whereas MC simulation should be used when the nature of the radiation and the tissue heterogeneities make this an essential part of the problem 5, 18, 66.

In a reversed version of the AD rate pipeline, first the AD is calculated via integration and then the AD rate 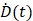 can be estimated at each time point, for instance, through DPK convolution and MC methods. The formula for calculating AD is as follows:


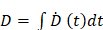
 (3)

This particular order is especially useful when the AD rate maps are generated directly by the technique (such as kernel or MC techniques), and it is commonly used in voxel-wise dosimetry protocols. In the following sections of the review, we will refer to the elements of the general protocol (acquisition, registration, activity/AD rate quantification, and summation) without any presupposition of a particular canonical order unless otherwise stated by a specific technique.

**Limitations:** The gold standard MC simulations take hours or days of computations, require advanced skills, and supercomputing facilities, making their practical application challenging in daily clinical practice 72, 73. Both the LED method and DPK method rely on assumptions that may be violated in reality (e.g., homogeneous tissue properties and local deposition), leading to inaccuracies in cases where there is heterogeneity of tissues or penetration radiation. This explains why AD assessment using DL (Section 4.6) and DT/PBPK models (Section 4.8) was proposed.

## 4. Approaches to Simplify Dosimetry Workflow to Overcome Challenges and Limitations

The conventional process for internal dosimetry was explained in Section 3, which also pointed out major drawbacks in it, such as MTP imaging, a laborious segmentation and registration process, computation-intensive AD calculation, and uncertainty in calculating TIACs. Simplification approaches described in this section were proposed with an intent to mitigate the aforementioned problems in mind. Hence, SPECT imaging time reduction and time-point reduction techniques can be regarded as simplification approaches dealing with the data collection problem, while automation of segmentation and registration steps is intended to minimize human involvement in the process; DL techniques are used to speed up computation-intensive radiation transport calculations; and PBPK, NLME, and DT approaches attempt to overcome the imaging problem using modeling.

The following discussion about simplification methodologies is valid for the numerous RPTs summarized in Table 1. For each approach, the current paper reviewed existing studies on validation of the specific approach through application to certain radiopharmaceuticals, which need to be considered more as an example than as a one-to-one pairing of the problem and its solution.

The development of a streamlined and minimally interactive workflow of patient-specific dosimetry in RPTs became particularly topical amid growing needs for the methodology among clinicians. Several approaches enabling to reduce the workload for medical staff while retaining dosimetric accuracy and clinical relevance have been suggested as part of internal dosimetry. The strategies include reduction of SPECT acquisition time, decrease in the number of imaging time points, automatic correction of SPECT data, segmentation, registration, AD estimation, prediction of AD, and computational modeling and DTs.

For each technique used for simplification, a systematic assessment is needed to address an important question: Has the simplified technique retained clinically adequate accuracy compared to a known standard of reference? Table 3 does this by assessing each simplification technique in terms of standard of reference, clinical challenges, quantifiable accuracy (bias, precision, limits of agreement) compared to the reference, validity conditions, and the technique itself. This table features eight simplification techniques (four from the STP subcategories and additional simplification methods). This section discusses each topic and addresses future directions.

### 4.1. Shortening SPECT Acquisition Time

Minimizing SPECT acquisition time for each bed position represents another simplification method. This technique makes patients more comfortable, which also enables to reduce motion artifacts. Yet, the acquisition process itself becomes less accurate, thereby directly impacting the accuracy of activity calculation, and hence also the AD estimation. Hence, this approach cannot be used by itself.

Research is now geared towards shortening SPECT acquisition time, simplifying dosimetry process to make it faster and more efficient without affecting accuracy. From studies validating accelerated SPECT acquisition in RPTs, two major strategies have been established that would help carrying out this task by compensating for short acquisition times: the enhancement of robustness of reconstruction at low-count levels and the compensation for missing projections. What distinguishes the two from each other is the way each strategy works. While the first uses acquired counts, the latter creates completely new projections.

Apart from the conventional techniques adopted in low-count imaging, novel approaches emerged for image reconstruction. One study looked into the effect of a reduction in frame time from 20 seconds to 10 and 5 seconds in [^177^Lu]Lu-PSMA dosimetry 74. For the quantification process, the same calibration factor was maintained throughout irrespective of the acquisition duration. Compared to the control group, which had a frame time of 20 seconds, the error percentages were 1.5%, 4.8%, and 1.4-3.2% in the case of the right kidney, left kidney, and tumor lesions, respectively. There was an increment in the relative percentage difference ranging between 2.9 and 6.6% for shorter frame times (5 seconds).

Salimi *et al*. 75 scanned a torso phantom using General Electric Starguide 360-degree CZT SPECT, reducing the acquisition time from 20 minutes to below one minute without compromising accuracy and obtaining recovery coefficients and gamma pass rates for lesions of various sizes to choose the best acquisition protocol and optimal acquisition time. The issue of low-count imaging is highly important when considering theranostics using radionuclides like ^177^Lu due to the low number of photons limiting the ability to perform dosimetry accurately. To solve this problem, DL techniques can be used to make the process more stable, providing a way to obtain accurate ADs using a shorter acquisition time. Specifically, Lim *et al*. 76 proposed using a trained regularizer during SPECT reconstruction of low counts combined with CT-based masks (nnUNet). With [^177^Lu]Lu-DOTATATE datasets obtained in humans and phantoms, they showed that their method allowed significant improvement in image quality, noise reduction, and quantitative accuracy compared to other regularizers.

Ryden *et al*. 77 suggested a U-Net architecture to decrease the acquisition time for ^177^Lu-SPECT scans by producing artificial intermediate projections with sparse data. Their model, trained on 352 clinical SPECT studies, was capable of producing an image quality comparable to complete-projection reconstruction, with only about a 2.5% difference in quantification of kidney activity. While supervised learning algorithms require labeled data, Li *et al*. 78 came up with a self-supervised algorithm based on a neural radiance field and coordinate-based learning that generates missing SPECT projections directly from the data without the need for training dataset. Using a multi-layer perceptron architecture, they could minimize scan time by up to 8× compared to linear interpolation and undersampling. In another approach by Nzatsi *et al*. 79, a Generative Adversarial Network (GAN) was employed to convert 6s images to artificially synthesized 30s images. This network was trained using phantom and clinical studies, enabling to generate organ-level activity in kidneys and liver with errors less than 6% and less than 1%, respectively, consistently showing better results than those obtained using 6s acquisitions when phantom tests were used. More importantly, it managed to cut down acquisition time from 45 minutes to below 10 minutes for three-bed SPECT imaging.

It is crucial to make another distinction, which is usually ignored. The issue of scan duration and time point count is two sides of one question. Decreasing the former is related to different conditions than decreasing the latter. There are two separate cases, namely: (1) decreased acquisition times lead to the possibility of performing the same number of time points but with increased comfort and decreased motion artifacts; and (2) shortened acquisition times increase the number of time points within the same total time burden, e.g., six scans of 10 minutes each instead of three 20-minute scans.

On the other hand, projection-synthesis techniques allow decreasing scan duration even further than reconstruction-based techniques, while the latter provides a better opportunity to control noise and perform quantification. In both cases, however, a general tendency can be noticed, namely: deep learning makes quantification reliable even when the counting level is considered too low by traditional standards. Nevertheless, some problems remain unsolved, such as cross-center validation and lesion heterogeneity.

### 4.2. Reducing Imaging Time Points

**Figure 4:**
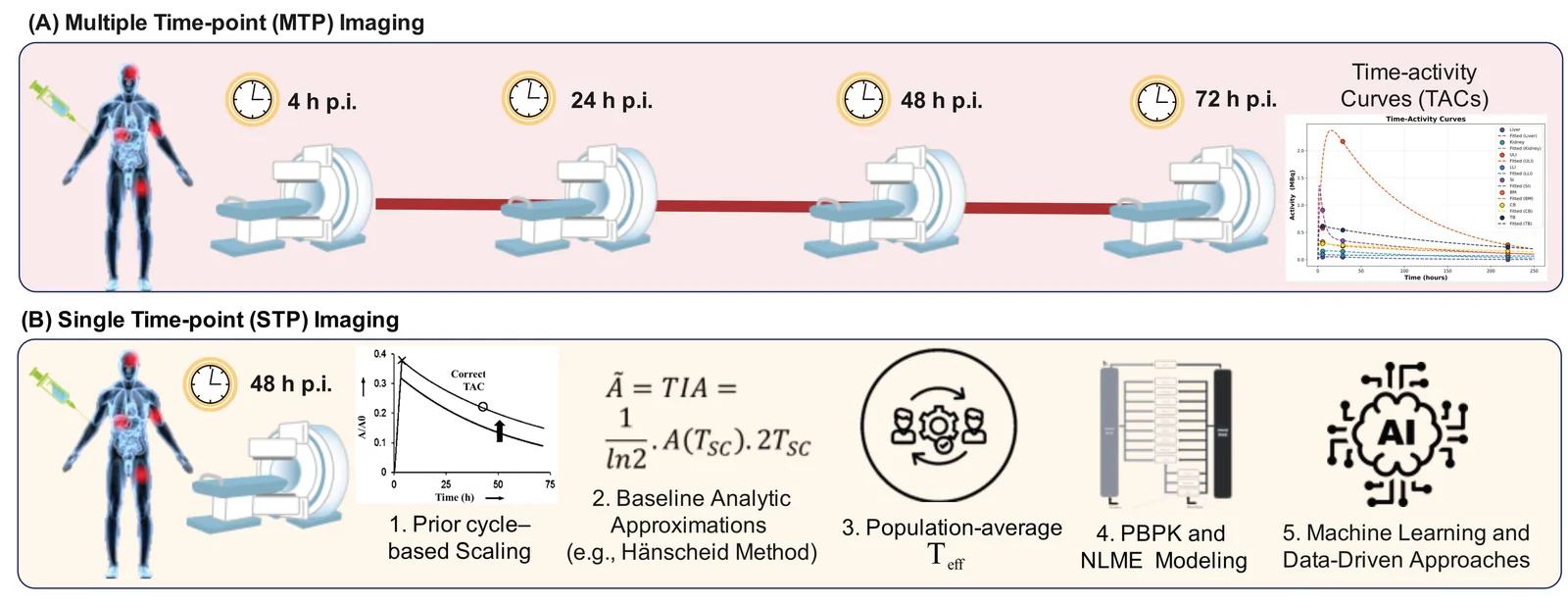
** (A)** The traditional multi-time-point (MTP) dosimetry approach relies on multiple scans to accurately measure the time–activity curves (TACs), but with significant clinical workload, while **(B)** the single-time-point (STP) techniques employ strategies such as: 1) prior cycle-based scaling, 2) baseline analytic approximations, 3) population data, 4) physiologically-based pharmacokinetic (PBPK) and nonlinear mixed-effect (NLME) modeling approaches, and 5) machine learning and data-driven approaches.

MTP imaging, with its complex nature and high workload, has been one of the barriers to the use of dosimetry for RPT. As can be seen in Fig. 4A, acquisitions, including those done at 4, 24, 48, and 72 h, etc., need to be performed to obtain a TAC fit and, consequently, TIACs, but not without affecting patients' compliance and costs.

Strategies allowing to solve these issues emerged thanks to the simplification of the STP dosimetry process. According to the diagram provided in Figure 4B, STP attempts to replicate the MTP dosimetry technique by utilizing only one image after the injection. It is necessary to mention that the ideal scanning time points depend on both the radiopharmaceuticals used and the method utilized, which may change due to the tissue studied. In terms of kidney dosimetry, the most efficient scanning times are considered to be around 72 hours in the case of [^177^Lu]Lu-DOTATATE versus 48 hours in the case of [^177^Lu]Lu-PSMA 80. Other STP methods rely on early scans (for instance, between 3 and 5 hours post-injection), using either population studies or machine learning 97.

These STP techniques may be further categorized into certain types depending on their assumptions. Some of the prominent STP techniques may be further grouped into five main categories. These include: (1) prior-cycle–based scaling, (2) baseline analytic approximations (e.g., Hänscheid *et al*. method), (3) population-average effective half-life (T_eff_) methods, (4) PBPK and NLME modeling approaches*,* and (5) primarily data-driven machine learning approaches.

#### 4.2.1 Prior-cycle-based Scaling

The assumptions made in prior-cycle scaling techniques are that the shape of the TAC curve, or biological clearance dynamics, is constant over successive cycles. In other words, the TIAC for the next cycle is calculated by scaling the TIA in the first cycle obtained using the MTP method with the ratio of one measurement per cycle relative to another 81. This is due to the assumption that the normalized shape of the TAC curve does not differ in successive cycles, only the amplitude does. The reference model in such cases is obtained via full MTP dosimetry conducted during the first cycle (usually 3-4 points in 4-7 days) 81-86.

As shown by Madsen *et al*. 82, with knowledge of the tracer kinetic information, estimation of the total integrated activity and AD through STP can be quite accurate. Strong correlations (R^2^>0.95) were observed between the measured and estimated ADs from [^90^Y]-DOTATOC.

A retrospective analysis by Kurth *et al*. 81 was conducted on two simplified dosimetric approaches to compare them with a complete imaging-based approach, namely M1 = [^177^Lu]Lu-PSMA-617 SPECT/CT at 2 h, 24 h, 48 h, and 72 h per cycle in 46 patients with mCRPC. In method 2 (M2), a cycle 1-based dose was applied for subsequent scans, and the most suitable one was 48 h. In method 3 (M3), ADs were determined through activity ratio-based extrapolation. The agreement of M2 with the reference was highest at 48 h but still underestimated the accumulated dose to the kidneys and parotids, more than M3. Likewise, Jackson *et al*. 84 standardized TACs on an STP scan performed for [^177^Lu]Lu-PSMA-617 treatment. They found that late scanning (>72 hours) is more accurate for tumor uptake ADs than early scanning (≤48 hours). Early scanning (≤48 hours), on the other hand, was more sensitive to normal tissue kinetics.

Karimzadeh *et al*. 85 studied the effectiveness of simplified methods (SMs) in the dosimetry of [^177^Lu]Lu-PSMA-I&T, through comparison to full dosimetry. They used three SM techniques, namely: (1) obtaining TACs for follow-up cycles based on STP imaging and assuming kinetics as per AD cycle 1, (2) extrapolation from AD cycle 1 alone, and (3) both AD cycle 1 and AD cycle 2. It was found that SM1 was effective in dosing OARs when performed via an STP image at 48 hours, while tumor dosing required STP images ≥ 72 h post-injection.

Ardenfors *et al*. 86 evaluated STP dosimetry for [¹⁷⁷Lu]Lu-DOTATATE and found that ADs for kidneys were the most accurate on day 1 and ADs for tumors on day 7 after therapy administration. Appropriate results were obtained using fixed or first-cycle-derived half-lives.

Such methods exploit the fact that the kinetics of organs can be similar throughout the cycles. Regardless of the type of radiopharmaceutical, the late STP approach, which is performed at radiopharmaceutical-specific late times (e.g., 48-72 hours for PSMA agents, enables consistent organ-level AD estimation and minimizes the necessity for MTP imaging in subsequent cycles 83-86. The term "MTP" used in the cited sources denotes a reference protocol involving 3-4 acquisitions using SPECT/CT over 4-7 days 83-86. For example, Kurth *et al*. 81 and Karimzadeh *et al*. 85 used four-time-point imaging as their standard of reference, while Jackson *et al*. 84 used three SPECT/CT scans (at 4, 24, and 72 hours) plus a planar scan at day 7. However, these prior-cycle scaling methods may underestimate tumor uptake in patients whose tumor biology changes more substantially between cycles. Thus, these techniques are suitable for use with organs having stable kinetics but not for heterogeneous tumor lesions 83, 84.

#### 4.2.2 Baseline Analytic Approximations (e.g., Hänscheid Method)

The Hänscheid technique is one such STP technique 87, which calculates ADs based on abdominal activity measurements, thereby lowering the patient load and complexity of the procedure. This particular technique rests on the premise that the scan takes place at a time when the correlation between the two can be approximated by:


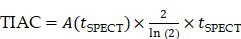
 (4)

which holds when imaging occurs between 0.75 and 2.5 times the effective half-life 87.

**Validation:** The approach was first validated for [^177^Lu]Lu-DOTATATE/DOTATOC radiopharmaceutical treatments, showing that AD map acquisition from one scan point at four days after injection is sufficient for acceptable accuracy 87. Further validation studies were carried out for different radiopharmaceuticals. The Hänscheid method was tested with different radiopharmaceuticals by Hou *et al*. 80, and it turned out that the best time to carry out the scans for [^177^Lu]Lu-DOTATATE and [^177^Lu]Lu-PSMA was 72 hours and 48 hours, respectively, after the injections for kidneys. However, the accuracy decreased when it came to [^177^Lu]Lu-PSMA, especially for the dosimetry of bone marrow and tumors 80. Chen *et al*. 88 compared the Hänscheid method against two-time-point approaches for [^177^Lu]Lu-PSMA, reporting that STP methods produced mean absolute errors of 8.05 ± 6.05% for kidneys and 6.14 ± 5.19% for tumors.

**Optimal timing:** Resch *et al*. 89 compared the efficacy of the Hänscheid method to other STP methods for [^177^Lu]Lu-PSMA-I&T therapy. They found that 48 hours post-injection is the optimal imaging time point for this method in targeting the kidneys. Nonetheless, for salivary glands, the Hänscheid technique was found to have reduced efficacy owing to biexponential kinetics, demonstrating the importance of kinetics in determining the accuracy of the technique 89.

**Limitations:** Several limitations of the Hänscheid method have been identified. First, Gustafsson and Taprogge 90 demonstrated that the Hänscheid method often produces negatively skewed error distributions. This method underestimates TIAs, particularly in patients with high biological half-life variability. Second, the method is sensitive to assumptions about biological decay rates. In this method, accuracy declines when the effective half-life deviates substantially from the population average 90, 91. Furthermore, the method assumes mono-exponential clearance, which may not hold for tissues with biexponential kinetics (e.g., salivary glands) or for tracers with more complex clearance patterns 92. Moreover, Peterson *et al*. 93 showed that the method performs adequately for organs with predictable kinetics (e.g., kidneys in DOTATATE therapy). Nevertheless, the method shows poor performance in case of tumors and tissues that exhibit high kinetic variability. The overall assessment regarding the Hänscheid technique is that it is an easy-to-apply and simple analytical procedure in STP, although caution needs to be exercised during application.

The comparison of reduced-time point or STP techniques against conventional full MTP references from the review of clinical validations over the past ten years involving different radiopharmaceuticals has resulted in categorizing STP techniques into five types (listed above). Other than specific STP techniques, several comparisons of the pros and cons of reduced time point versus complete MTP techniques have been performed by other groups 80, 87-89, 91-93. Hou *et al*. 80 found the average percentage error in TIAC to decrease for STP techniques from 20-30% to 10-15%. The work done by Peterson *et al*. 93 proved that three-time-point schedules lowered the uncertainties in TIAC to a range between 5% and 10% when compared to the 4-5 time-point schedules (MTP). Resch *et al*. 89 observed that a two-time-point schedule (48 h + 96 h post-injection) obtained the kidney ADs at a difference of 5% from MTP and ADs at tumors within 10%-15% for [^177^Lu]Lu-PSMA therapy. Hence, the reduced time point technique provides a balance between accuracy and the low workload of MTP techniques.

#### 4.2.3 Population-Average Effective Half-Life (T_eff_)

There is another group of STP methodologies that uses population-average effective half-lives or simplified analytical formulas for estimating TIA based on one activity value 89, 92, 94. Concerning [¹⁷⁷Lu]Lu-DOTATATE RPT, Vergnaud *et al*. 94 employed a simplified dosimetry protocol based on the tri-exponential curve fit obtained in the first cycle (approximately 1, 24, and 96/144 hours), complemented with a single measurement at 24 hours in subsequent cycles. If there were no 24-hour values available, a population-based model (STP-Inter) was used. In relation to the STP-Intra approach tailored for the individual patient, deviations in percentage AD at 24 hours for the left kidney, right kidney, liver, and spleen were 0.7%, -0.2%, -0.5%, and 1.4%, respectively. In reference to the use of a later time period (96-144 hours/7 days), deviations were between -1.2% and 5.4%. For the population-based STP-Inter method, median deviations were < 7.7% for all volumes.

Resch *et al*. 92 investigated optimal time samplings for [¹⁷⁷Lu]Lu-PSMA-I&T. For kidneys, three time points (days 1,2,3) achieved a mean deviation of 0±1% from the four-time-point reference. For lesions, three time points, including a late scan (days 1,3,7), achieved 4-5% deviation, with 95% of lesions showing < 10% deviation. However, using only early time points (days 1 and 2), deviation was 49±96%, with only 37% of lesions within < 10% 86. In a separate study, Resch *et al*. 89 demonstrated that lesion effective half-life variability (mean 56.5 ± 18.8 hours; CV = 33.2%) was substantially higher than kidneys (32.9 ± 5.9 hours; CV = 17.8%) and salivary glands (22.5±2.8 hours; CV = 12.3%). Based on these quantitative findings, population-averaged methods perform "well" for organs with low kinetic variability (CV < 20%). These methods are "practical" for routine organ dosimetry. However, for lesions with higher variability (CV > 30%), they might be "insufficient" for precise lesion-level estimation.

#### 4.2.4 PBPK and NLME Modeling Approaches

Advanced techniques for STP include approaches that rely on pharmacokinetic modeling through PBPK and NLME modeling schemes 90, 95-98. In addition, these models take into consideration uptake and clearance of the drug in the body, thereby making it possible to sample the analysis at optimal times.

The mechanistic edge that PBPK and NLME approaches have over simpler analytical formulas is the capacity to conduct inference based on scarce data by utilizing physiological prior knowledge 99, 100. In contrast to a simple equation (e.g., Hänscheid method) where the correlation between a single measurement and TIA is assumed to be constant, PBPK models incorporate physiological correlations (organ perfusion, ligand-receptor kinetics, clearance rates) by expressing them through systems of differential equations 99, 101. Having one measured value allows estimating physiological values using these equations with the help of the existing biological constraints. Likewise, NLME models combine population-based data to estimate fixed effects and random effects, which allows making inferences about particular patients even with a single measurement for each patient 100. The ability to conduct inference cannot be obtained by simpler analytical methods since they only interpolate and extrapolate values.

There is a clear difference between PBPK modeling and the NLME approach since these methods have different issues in the field of pharmacokinetics. PBPK modeling is defined as a particular structure of a model in which various physiological compartments of the body (such as blood, organs, and tumors) are connected with parameters representing blood flow and permeability through a physiological mechanism. The structure of the model, which involves the choice of compartments and connections among them, is decided upon beforehand based on the knowledge regarding the physiological characteristics of the behavior of the radiopharmaceutical. On the other hand, NLME stands for a particular type of fitting approach used to estimate both population-related parameters (fixed effects) and individual variability (random effects) within one technique. NLME can be applied to any kind of model structure and kinetics, such as empirical exponential curves, compartmental, and PBPK models 90, 95-98.

It can be inferred from literature studies that model-based methods usually produce better TIAC and AD values than analytical methods under certain specific circumstances. Hardiansyah *et al*. 98 described population-based NLME and PBPK modeling for STP dosimetry in [^90^Y]Y-DOTATATE PRRT using [^111^In]In-DOTATATE data from eight patients. TIACs and ADs were estimated for several organs and tumors using MTP method and compared with the results obtained from a single value collected at around 47 hours post-injection. Both kidney and tumor dosimetry produced the best results in terms of accuracy using the PBPK/NLME-based STP approach, equaling the standard sum-of-exponential methods.

Hardiansyah *et al*. 95 used model selection within the NLME framework (MS-NLME) to examine the [^111^In]In-DOTATATE biokinetics in the kidneys, based on their previous study. For fitting biokinetic data from all eight subjects, eleven functions using mono-, bi-, and tri-exponential models were used, and the Akaike weight was used for choosing the best fit. Four-parameter functions gave the best support in this regard. The MS-NLME method has proved to be effective in calculating TIAs in STP dosimetry irrespective of the radiopharmaceuticals used and the patients studied. In another study carried out by Budiansah *et al*. 97, PBPK model parameters were optimized through the use of extra variables. According to this study, the use of 48 h p.i. was proposed as the most appropriate STP. While accuracy was acceptable (7% for kidneys, 14% for tumors), STP dosimetry was less precise than all-time-point (ATP) methods.

In a study comprising 63 patients with mCRPC undergoing treatment with [^177^Lu]Lu-PSMA-617 RLT, Hardiansyah *et al*. 96 applied the population-based model selection (PBMS)-NLME for renal dosimetry analysis based on SPECT/CT scans. Thirteen functions using one- to five-exponential modeling were fit against ATP data, and among these functions, the function that provided the best fit was selected by the PBMS NLME method. At the same time, STP data of an individual patient were fit against ATP data of all other patients using the PBMS NLME method. The PBMS NLME showed superior results compared to the Hänscheid and Madsen methods, with the smallest RMSE value at point 3 (42.6 h).

To evaluate the effect of inaccuracies in estimating activities on the spread of TIA during RPT dosimetry, a theory has been put forward by Gustafsson and Taprogge 90. The theoretical formulation is based on applying the law of propagation of uncertainties to arrive at expressions for the variances of relative errors and TIA differences with time points removed. The theory was validated for 18 patients undergoing therapy with [^177^Lu]Lu-DOTATATE, along with numerical verification. It was found that the TIA spread would be minimal with time points taken around the mean of 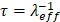.

The model-based STP methods are shown to significantly outperform simple analytic expressions, particularly when there are large variations in biological half-life among subjects. They are more robust when it comes to various tracers and target organs. The main difficulties in their application are connected with the necessity to calibrate the model (estimation of patient-specific parameters, e.g., association rate, internalization rate, and receptor density based on limited data), the use of population priors (population data is necessary in the case of the Bayesian estimation approach used in NLME modeling that may not be easily available); and computation-intensive solving of ordinary differential equations 100, 101.

One basic methodological issue that arises in relation to studies of STP dosimetry techniques is structural model misspecification when validating simple dosimetric techniques. Indeed, a large number of studies conducted on STP have relied on mono-exponential modeling of TACs as the basis for TIAC calculation. The problem here is that mono-exponential modeling can be considered inappropriate in cases where multi-exponential modeling needs to be used for radiopharmaceuticals having rapid distribution and slow clearance. In other words, even when an agreement has been found between STP and mono-exponential modeling, the findings do not provide a basis for validation of accuracy since they both may be misspecified models 90, 95. However, a much more robust and defendable alternative is to explicitly take model uncertainty into account, instead of relying on the fixed functional form assumption 95, 96. The rationale behind such an assumption is that different subjects, different organs, different tracer agents, and the number of imaging time points available, might need to use different models.

From the perspective of methodological credibility, PBPK models, and especially those utilizing the NLME approach, are endorsed by both the FDA and the EMA as part of their official standards when it comes to drug development and radiopharmacokinetic studies 102. Such techniques are exceptionally useful when dealing with sparse data sets and disparate sampling procedures, issues which are often encountered during RPT dosimetry. This approach creates the necessary groundwork to justify the transition from NLME techniques to clinical dosimetry procedures and thereby validate the use of PBMS-NLME standards as STP dosimetry techniques.

Besides robustness in general, there are three clinical applications for which model-based methods can be used that cannot be solved analytically 99, 100. The first one deals with predicting the activity in compartments or organs in which measuring the activity is impossible or inaccurate (bone marrow, small metastases, and superimposed uptakes) using measured activity in measurable organs to define the whole system 99. Second, they provide an opportunity for interpolating the value of the parameter under consideration when its measurement becomes impossible due to illness or other factors. In particular, the case in which the patient fails to show up for the appointment with his/her scan will be predicted by the use of PBPK/NLME models based on information provided by the population as well as by measurements made on the patient himself, which is not possible in the case of analytical formulas 100. Finally, they provide possibilities for prospective planning of therapeutic interventions through simulation of effects that different administration activities would have on ADs 99-101.

In describing the performance of STPs, it is essential to clearly identify the reference standard that was used in each study. Concerning these points, it is proposed that any future validation work for STPs consider reporting: (i) the form of the reference standard that was assumed, (ii) if there was model selection or if there was forcing of the form chosen, (iii) the criteria that were used to select the form of the model (Akaike Information Criterion, Bayesian Information Criterion, or Akaike weights), and (iv) in cases where they exist, if the procedure conforms to recognized regulator population modeling techniques.

#### 4.2.5 Primarily Data-Driven Machine Learning Approaches

Other novel techniques, besides parametric modeling and PK modeling, involve the application of either generalized additive model (GAM) or ML to predict kinetic parameters through imaging or baseline biomarkers 103, 104. Such methods can account for the presence of nonlinearity and individuality better than any conventional analytical methods, which, in the current case, include (i) multi-exponential fitting (fitting mono-exponential functions to time-activity curves); (ii) population-based strategies such as that used in the approach by Madsen *et al*. 82 (population average effective half-life); and (iii) approximation strategies including the Hänscheid method. Traditional analytical methods cannot account for interpatient variability because they apply a fixed form of analysis across all patients without incorporating patient information and learning from the available data to correct individual kinetic variations. Data-driven models have proved better when compared in terms of mean absolute error (MAE) by applying leave-one-out cross-validation: while estimating the kidney TIA at TP1 (3-5 hours post injection), Wang *et al*. 104 proposed the use of the GAM approach to reduce the MAE of the method from 15.6% (using the Madsen method) to 11.8%; whereas at TP2, the use of GAM reduced the MAE to 8.7% compared to 20.7% achieved by the Hänscheid method.

Initial results for [^177^Lu]Lu-DOTATATE RPT show that including a single activity measurement using SPECT at time point 1 (3-5 hours after injection), together with the relevant biomarkers, improves prediction compared to approaches based solely on the single activity measurement (such as the Madsen or Hänscheid methods). For instance, when the glomerular filtration rate (eGFR) was included as a biomarker to predict renal function for the kidneys, the MAE decreased from 15.6% ± 1.3% to 11.8% ± 1.0%. Including the tumor volume as a biomarker to estimate tumor function lowered the MAE from 30.5% to 27.1% 104. It is worth mentioning that the inclusion of these biomarkers showed their benefits only for TP1 and not for later time points (days 1-8).

In totality, the progress made in the domain of reduced-time-point dosimetry has evolved from mere mathematical analysis of STP to more advanced approaches. Although there is no one best strategy for all scenarios, in general, a well-timed STP or TP method could provide sufficient dosimetry in clinical terms for many organ systems and tracers 7, 8, 104. Nevertheless, accuracy is still case- and tracer-specific, especially when the kinetic behavior of the tumor/tissue under study is not homogeneous.

### 4.3. Automated SPECT Data Corrections

Data correction is an essential requirement for quantitative SPECT and continues to be important for accurate dosimetry in RPT. Existing pipelines rely upon CT-based attenuation and scatter corrections, resolution modeling, and calibration based on phantoms; all of which carry with them issues related to added complexity, sensitivity to misregistration, and potential for error propagation through the dosimetry chain 5. DL advancements have allowed for automatic correction approaches which not only save on computation but also prevent artifacts and may even improve accuracy. Despite advances showing strong promise for theranostics, for example, [^177^Lu]Lu-PSMA and ^90^Y radioembolization, and also pretherapeutic imaging with ^99m^Tc-MAA, several uncertainties need to be addressed, including generalizability from one machine to another, dependency on large training sets, sensitivity to out-of-distribution anatomy, and overfitting 105-111. Comparison of conventional methods versus DL correction techniques is provided in Table 4.

#### 4.3.1. Attenuation Correction

Quantification is particularly vulnerable to the presence of artifacts due to photon attenuation effect. Although the conventional CT-based attenuation correction (AC) procedure is adequate in this case, it has drawbacks, such as the misalignment of SPECT and CT images as well as artifacts generated due to the motion of the subjects being examined. New DL techniques overcome these problems by modeling the connection between raw images and AC images through either μ-map synthesis or image-to-image learning approaches 106, 112, 113. These approaches do not need CT scans since there is no misregistration and related artifacts. It should be noted that DL techniques give smoother quantification of the activities, especially in regions where there are issues with CT images or misregistration.

Gehring *et al*. 122 explored the use of DL to achieve AC without the use of CT images for ^177^Lu-SPECT studies, with the aim of lowering radiation dose to patients and preventing possible errors in image alignment. The training of the 3D U-Net model was first performed using the 10,000 simulated datasets and then fine-tuned using over 1,000 sets of clinical SPECT/CT images obtained from patients receiving ^177^Lu treatment. They managed to obtain AC SPECT images from non-AC SPECT images through DL. The image quality was assessed using three metrics, namely structural similarity index measurement (SSIM), normalized root mean square error (NRMSE), and volume activity accuracy (VAA). It was shown that the DL-based approach to AC performed better than conventional non-AC reconstruction techniques by obtaining CT-like correction of images.

#### 4.3.2. Scatter Correction

Traditionally, scatter correction (SC) can be carried out using energy windowing techniques or model-based methods. MC modeling, which belongs to the second category, is very accurate but is computationally intensive. The goal of DL-based scatter estimation models is to replicate MC-level accuracy while greatly reducing computation time. Convolutional neural networks (CNNs) or transformer-based approaches can perform SC alone or both attenuation and scatter corrections together, facilitating a trend towards an integrated approach to correction 108, 109, 115, 116, 123. Moreover, an important point here is shifting from correction modules that operate independently to DL methods trained using large amounts of data consisting of simulated or real scans of patients. Such an approach appears to be highly promising when used for ^90^Y and ^177^Lu dosimetry since scattering is responsible for nearly most quantification errors in this case. However, generalization of these methods is still to be tested multicentrically.

Jia *et al*. 108 developed a DL algorithm for ^90^Y SPECT scatter correction and dose calculation in liver radioembolization characterized by low spatial resolution and complex scattering pattern. The DL approach includes a CNN for scatter estimation and DblurDoseNet for AD rate computation, where the training data included virtual patient phantoms and MC-based ground truth. When compared to MC dosimetry, the approach showed improvement in normalized mean absolute error (NMAE) in lesions from 24.0% to 8.6%, provided better lesion delineation, and produced 3D AD maps within ~20 s with GPU usage. Hence, this method demonstrated higher accuracy and efficiency for clinical dosimetry purposes. MC SC can be applied for better scatter and dose modelling for pure beta emitters, such as ^90^Y. Fortunately, such tools are available on commercial software (Hermes^1^) and open-source software (PyTomography^2^). According to Mansouri *et al*. 109, transformer-based DL algorithms were capable of calculating MC SC and CT-based AD for average AD rates and distribution in lesions and OARs with gamma pass rate analysis.

#### 4.3.3. Partial-Volume Effect (PVE) Correction

Due to the low resolution of SPECT images, PVE occurs, resulting in underestimated activity concentrations in small lesions and heterogeneously distributed organs 105, 124. Hence, improving spatial resolution is the key task for further investigations 107. In therapeutic imaging, e.g., [^177^Lu]Lu-PSMA therapy, accurate partial volume effect correction (PVC) becomes necessary to accurately quantify uptake, perform proper dosimetry, and plan adequate therapy. Traditionally, PVC can be achieved either by recovering coefficients or segmentation approaches, which are prone to noise influence and alignment errors 117, 125. DL-based PVC techniques require large training datasets using MC simulations or specific phantom studies to recover the resolution, irrespective of the manual segmentation procedure 105, 110, 117, 118. These methods turned out to be better than others for the recovery of the tracer and artifact reduction, which is very important in dosimetry applications.

Concerning AC, SC, and PVC, there appears to be a trend towards the development of DL algorithms employing end-to-end learning and reduce the number of independent calibration and preprocessing procedures required for quantitative SPECT imaging. Instead of specifying individual correction procedures, recent literature has attempted to learn the joint correction procedure from preprocessed or even raw images 109, 115, 116. The idea of simplified dosimetry is also reflected in this approach, as minimal operator intervention, lower amount of scanning procedures, and increased stability between centers are highly desirable. Nonetheless, one has to admit that the performance of DL methods that learn one function is superior to the performance of methods that simultaneously learn multiple functions. However, implementation of this method into clinical practice demands thorough testing of the method's performance to provide sufficient proof of robustness regardless of scanners, protocols, and radiopharmaceuticals used.

Leube *et al*. 110 investigated the DL-PVC approach in relation to ^177^Lu SPECT/CT imaging. DL-PVC was trained on 10,000 phantom images generated through MC simulations, achieving an SSIM of 0.95, NRMSE of 7.8%, and VAA of 35.8%, which is better than results obtained with non-PVC SPECT imaging (SSIM 0.89, NRMSE 10.4%, VAA 12.1%) and iterative Yang PVC (SSIM 0.94, NRMSE 8.6%, VAA 15.1%). The use of 3D-printed phantoms also demonstrated the capability of DL-PVC to produce accurate recovery of activity without segmentation while correcting for Gibbs artifacts.

PVC techniques can utilize structurally co-registered images, in this scenario CT images, to rectify the problem of the spill-in/spill-out phenomenon. Nevertheless, potential misregistration between SPECT and CT images, which is prevalent, particularly in organs involved in respiratory motion like the lungs and liver, may lead to erroneous results.

### 4.4. Automated Segmentation

The manual segmentation performed by a skilled operator is used as benchmark against which different techniques can be compared in terms of tumor or organ delineation. To measure inter-operator agreement, segmentations performed by several operators should be considered, along with repeat segmentations performed by each operator in the case of inter-operator variability assessment. With respect to dosimetric applications, OARs are typically segmented from SPECT/CT images based on the information obtained from CT images, with adjustments made to compensate for organ movements. Delineating tumors on non-contrast-enhanced CT images with low contrast properties proved to be difficult. As such, it is preferable to use SPECT or pre-therapeutic image segmentations 5. SPECT segmentation is challenged by noise, low resolution, PVEs, and spill-out effects, leading to activity misplacement and underestimation in high-activity regions, affecting internal AD assessments.

Nevertheless, conventional methods, thresholding-based methods, and DL-based methods have been investigated for improved segmentation accuracy 126. Most of these approaches depend on hardware, scanning procedures, and image reconstruction. The deployment of such tools in different clinical centers is complicated without proper validation. To solve this problem, Task Group 211 of the American Association of Physicists in Medicine (AAPM) provided a set of criteria for evaluating the performance of PET segmentation algorithms, such as accuracy, precision (which includes reproducibility and repeatability), efficiency, and robustness 127. Moreover, some applications for PET imaging are similar to those of SPECT. Hence, they might be applied to SPECT segmentation as well 127.

#### 4.4.1. Classical and Thresholding-based Methods

Thresholding-based methods remain the most approachable segmentation methods in SPECT 126. Adaptive and data-driven thresholds proved to outperform fixed thresholds and iterative algorithms, especially those based on recovery coefficient modeling, providing augmented robustness to noise, lesion size, and reconstruction parameters 128-131. In general, however, classic thresholding methods are computationally efficient but are affected by image characteristics and lesion properties.

#### 4.4.2. Deep Learning for Organ and Lesion Segmentation

DL techniques have gained prominence in the automatic segmentation task within the realm of internal dosimetry, spurred by their effectiveness in organ contouring for external beam radiation therapy (EBRT) 132 and, more recently, in nuclear medicine 133. DL methods have been implemented for segmentation of organs in CT 134, 135 and PET/CT imaging 136-138. Lesions are much more challenging to outline compared to organs due to their large variability in terms of shape, size, and anatomic positioning. For RPT, accurate segmentation of lesions is critical to obtain clinically meaningful biomarkers and quantify disease burden. Volume-based biomarkers obtained through AI-driven segmentation are directly related to tumor burden, overall survival, and prognosis 15. CNN models have demonstrated similar performance compared to human experts at organ and lesion segmentation tasks using 2D, 3D, and U-Net approaches 139-143. DL models trained using large or diverse training sets tend to perform better than those with small amounts of training data. In addition, models that include preprocessing or use multi-modal inputs, such as fused SPECT/CT, improve the accuracy of these methods, especially when applied to small or heterogeneous structures. All in all, the application of these DL methods has enabled accurate organ and lesion delineation for organ and voxel-level dosimetry in research and clinical settings.

The challenge in metastasis detection in PSMA PET/CT imaging arises due to physiological uptake, leading to a shift in the AI approach for identifying metastases instead of primary tumors and providing full planning for RPT 137, 143, 144. In NETs, the precise delineation of lesions on ⁶⁸Ga- and ⁶⁴Cu-DOTATATE PET/CT identifies tumor load and tumor heterogeneity, with an excellent agreement of AI-based contouring with expert-contoured regions 145-147. Despite the success of DL models in producing high-accuracy results for PET segmentation tasks, they have found limited applicability in SPECT image analysis owing to issues related to lesion growth/atrophy, deformation, and shifts in location. The problems have been further exacerbated by the lack of annotated datasets and the poor generalization capability of such models 148. Other techniques have been designed specifically to address aspects unique to SPECT; for instance, fuzzy C-means (FCM) clustering takes into account both the intensity of voxels and their spatial relation, thus making it a better choice for use with SPECT images with their fuzzy borders 126, 148. An X-means clustering technique for bone-marrow segmentation on [^177^Lu]Lu-PSMA-617 SPECT/CT was developed by Lu *et al*. 149, which proved to be more consistent and faster than manual outlining.

Existing algorithms require a huge number of labeled datasets to train, which is difficult to achieve in the medical imaging field due to issues related to annotations and patient privacy. The solution to such problems can be found in self-supervised learning (SSL), where learning takes place without any supervision using unlabeled data in applications, such as image reconstruction, assisting with image segmentation, and even classification 150. It was observed that pretraining the transformer model on large volumes of unlabeled PET/CT images followed by fine-tuning on a small labeled dataset could result in better results than traditional nnUNet for the segmentation of organs and lesions in PSMA imaging 137.

### 4.5. Automated Registration

Image registration is an essential process in personalized dosimetry for RPTs. Image registration is involved in two steps: (i) SPECT/CT image registration for correcting any motion artifacts, and (ii) registration between different sets of serial images, such as SPECT/CT or planar images. Any errors occurring due to misregistration, even a small error, can cause significant bias in voxel level dosimetry; hence, the need for proper automation in motion correction algorithms 151, 152.

Recent studies seem to indicate that certain trends exist in the automation of the registration process. To begin with, it should be noted that there is significant room for improvement of tracer uptake and AD estimation owing to misregistration between SPECT and CT images, which may be the case for small and highly movable structures 151. Techniques using both anatomical and functional data are quite resistant to temporal aspects and are applicable in pharmacokinetic models of small organs 84. Deformable image registration became increasingly popular as it achieves improved performance on serial images and deformed structures, such as the head and neck, shoulders, and thoraco-abdominal regions. Rigid registration algorithms may be successful for static structures; however, not all of them are, thereby calling for the application of deformable registration algorithms 152. DL-based registration methods are more attractive in terms of speed and robustness. In this context, an advanced method, called CoRX-NET, has been developed, where unsupervised DL models enable effective registration of images presenting with deformable motion patterns, thus improving TIA maps and decreasing operators’ intervention 153. Finally, workflow optimization and clinical feasibility issues have been pointed out. For example, automated co-registration methods have been studied to demonstrate that simplifying the workflow does not affect the accuracy 154. In this regard, contour-based automated registration provides a high-quality registration of image series, hence improving the accuracy of dosimetry calculations 45.

In conclusion, there has been an evolution from the first approaches used in rigid and manual registration to automated and deformable models employing AI. Future research should concentrate on standardizing this methodology across different centers and testing DL algorithms in different RPTs.

### 4.6. Automated Absorbed Dose Estimation

The ability to accurately estimate AD is important for planning individualized RPT strategies. However, conventional dosimetry approaches tend to make simplifying assumptions about the homogeneity of tissue and uniform anatomical modeling. Although MC simulation continues to be regarded as the gold standard for dosimetry, its computationally intensive nature restricts its routine use in clinical practice. The recent progress in AD prediction using DL constitutes a definite methodological evolution toward the level of accuracy achieved by MC simulations 142, 155, 156.

Initially used in internal dosimetry, DL algorithms were utilized to improve the MIRD methodology by introducing anatomical and density data specific to each individual patient. Due to the introduction of tissue heterogeneities into the kernel calculation, it was possible to improve agreement with MC calculations compared with homogeneous phantoms 157. These results show that anatomic differences should be considered for more accurate dosimetry.

Future studies focused on hybrid methods combining DL with conventional kernel and/or decomposition methods. Instead of completely ignoring prior methods, DL was used to refine intermediate steps like those involving features extracted from densities or AD components, hence resulting in higher accuracy for regions containing complex tissue boundaries or density differences 155, 156. While the approaches rely on the use of kernel methods, it becomes clear that it is possible to utilize DL in dosimetric studies with the help of the former approach without relying solely on the learning paradigm. The successful application of the above-mentioned approaches allowed switching to direct voxel-wise AD estimation. It has been shown that the use of CNN models fed with paired data of activity distribution, density images obtained by CT, and MC-generated ADs can learn the voxel-based AD estimation procedure without computing kernels 158.

More recently, voxel level DL approaches attempted to address problems associated with image resolution and classical modeling. In this regard, the application of residual learning techniques has been suggested as a way to correct SPECT-based AD estimates suffering from blurring/noise using DL to model the difference between classical reconstruction approaches and the AD map derived using high-resolution MC simulations 159. These physics–DL hybrids aim to achieve an interpretable solution while considering effects that cannot be accounted for analytically or with image-based methods.

A new dosimetry technique was proposed by Götz *et al*. 155 consisting of combining deep neural networks and empirical mode decomposition (EMD) to enhance the accuracy of AD estimation. Using CT density maps and MC simulations, it was possible to acquire reference values of ADs in different tissues of 26 patients treated with [^177^Lu]Lu-PSMA RPT reporting significantly higher accuracy and stability than the classical MIRD method. Furthermore, Götz *et al*. 156 estimated dose voxel kernels (DVKs) in kidneys, constructing a neural network based on 52,274 density kernels together with MC DVKs obtained for 26 patients receiving either [^177^Lu]Lu-DOTATOC or [^177^Lu]Lu-PSMA treatment. The findings showed that the proposed method was more efficient than the classical method using homogeneous kernels for convolution.

In Akhavanallaf *et al*. 157 a DL-based framework was used for the estimation of organ-level dosimetry in a subject-specific way at the whole-body scale using data obtained from dynamic [^18^F]-FDG PET scans of 24 patients. This technique involved training a DNN using density maps extracted from CT and S-values obtained from MC simulations to estimate specific S-value kernels. These kernels were then applied to the activity map to obtain AD distribution maps, in compliance with the MIRD formalism. The proposed approach showed good agreement with MC simulations, with an MRAE of 2.6%, thus outperforming other methods, such as the MIRD-based approach and OLINDA/EXM.

In addition, Li *et al*. 159 designed a residual CNN model (DblurDoseNet) for performing real-time, voxel-wise dosimetry calculations from [^177^Lu]Lu-DOTATATE SPECT/CT imaging. Specifically, the proposed neural network model was constructed by training it using dose rate maps (DRMs) generated by MC simulations on nine virtual patient phantoms. The evaluation of the network's performance was performed on five additional virtual patient phantoms and 42 patients, thereby allowing training to compensate for the effect of SPECT resolution. When compared to conventional DVKs and MC simulation approaches, the CNN model produced remarkably lower mean AD rate error (by 53%–55% for lesions and 56%–66% for kidneys) and NRMSE, as well as higher accuracy in simulating noise and AD volume histograms. The method provides generation of full 3D DRMs within 30 seconds, which demonstrates the ability of accurate and fast dosimetry.

On the other hand, combined pipeline approaches incorporating segmentation, registration, and AD-rate calculations have been proposed. This approach demonstrates the ability to achieve accuracy on a voxel-by-voxel basis without an increase in manual effort, akin to MC dosimetry methods 142. It should be noted that DL-assisted voxel dosimetry is effective regardless of the radionuclide used for treatment. Apart from being applicable in SSTR and PSMA treatments, other use cases involving radioactive iodine treatment and transformer-based AD enhancement methods demonstrated modality independence of such approaches 160, 161.

An automatic procedure for conducting clinical dosimetry at the voxel level in [^177^Lu]Lu-DOTATATE RPT has been developed by Dewaraja *et al*. 142. It consists of the following steps: organ segmentation using a CNN, intensity-based SPECT image registration according, MC-based computation of the AD rate, and fitting the curve at the voxel level. Using this approach, twenty patients with four-point SPECT/CT images have been analyzed, demonstrating high accuracy in organ segmentation (Dice coefficients ranging from 0.91 to 0.94 for kidneys), efficiency of the processing time (< 25 mins; about 2 minutes of manual work included), and high variability in ADs and number of cycles between patients.

Georgiou *et al*. 160 introduced a novel technique for dosimetric analysis of radioactive iodine treatment (RAIT) for differentiated thyroid carcinoma (DTC), which is suggested to facilitate the existing MIRD approach. They examined the applicability of their DL model to 83 patients, where the maximum permissible activity (MPA) may be determined using data at earlier time points (4, 24, and 48 hours), that does not differ significantly from the classical one (p = 0.351).

Mansouri *et al*. 161 devised a hybrid transformer-based DL algorithm using the UNETR framework for voxel-wise dosimetry of [^177^Lu]Lu-DOTATATE treatments. The methodology of multiple S-value (MSV) was adopted, and the DL model was trained to predict MC-based AD maps from CT images via learning the difference in results between MC and MSV methods. Validation on 22 patients during 4 therapeutic cycles demonstrated that the DL algorithm yielded better accuracy (voxel RAE = 5.28 ± 1.32) with a higher gamma pass rate (99.0 ± 1.2%) than both MSV and SSV techniques in only 3 seconds per scan.

The gathered information clearly demonstrated the direction towards which the automation of RPT dosimetry should proceed, that is, from DL-based corrections at the organ scale to physics-based AD estimates at the voxel level. This approach does not exclude MC simulations but strives to reproduce its results under quasi-real-time conditions for the sake of individual patient dosimetry.

### 4.7. Absorbed Dose Prediction

Apart from the assessment of ADs retrospectively, a number of studies have been conducted to predict ADs based on pre-therapy imaging and clinical parameters. It has been observed across all PET scans using either SSTR-targeted or PSMA-targeted radiopharmaceuticals that pre-therapy PET parameters are predictive of AD, especially for OARs 51, 162, 163. While the correlation between tumors is not very strong, these studies have shown the predictability of PET SUV biomarkers.

ML and DL approaches improve the relationship between dose parameters and PET attributes through clinical biomarkers (CBs) or radiomics. As stated by Xue *et al*. 164, personalized dosimetric parameters for [^177^Lu]Lu-PSMA treatment for patients could be predicted using pretreatment PET/CT and CBs via ML models, which outperformed population dosimetry. In continuation of the above-mentioned approach, some studies used radiomics, dosiomics, and CBs to predict the organ- and lesion-level ADs, while a transformer-based neural network was utilized to map AD rate voxel-wise with good correlation with MC simulations 50, 165, 166.

Additionally, the application of predictive modeling has been applied in liver radioembolization and thyroid cancer, where the surrogate from before treatment was able to predict the ADs post-treatment (for example, MAA SPECT or early time points) 167, 168. For PRRT, the use of radiomics and dosiomics with ML algorithms provides a good basis for accurate prediction of the ADs of organs 49, 52, 169, 170.

In addition to the predictive models based on ML and data analysis, there is another group of action mechanisms based on pharmacokinetic predictions, such as PBPK and NLME models. Siebinga *et al*. (2022) 16 reported that PBPK models integrate knowledge on drug properties and physiological parameters, thereby enabling a reliable estimation of whole-body distribution, while NLME models contribute to patient variability assessment. Both methods proved useful in estimating the transition from pre-therapy dosimetry to post-therapy through consideration of the peptide quantity, affinity of receptors, and rate of peptide internalization, which cannot be achieved through simple SUV correlation. The advent of PBPK-based STP models has resulted in more accurate estimates of AD for kidneys and tumors than empirical formalisms, while PBPK-DL model architecture helps in predicting AD on a voxel basis from pre-treatment images.

From the above discussion, one can conclude that the trend is now moving towards developing data-driven models based on personalized pre-therapy dosimetry.

### 4.8. Computational Modeling and Digital Twins

Before proceeding to discuss how DT and PBPK models are simplifications, it is important to first understand what is meant by "simplification”. During this review, simplification shall refer to any reduction in clinical burden, in imaging time points, less human intervention, and in the amount of data required. Thus, although these models may seem complex, they can still be regarded as simplifications in that they make it possible to perform dosimetry with minimal data required (STP/pretreatment imaging). This is an important clarification in the sense that even though some methods may entail considerable amounts of computations and data gathering, the overall result could lead to simplification.

The development of a DT is a time-consuming process, including such features as infrastructure, multi-modal data collection, and computations (methodologically complex). Nevertheless, after development and validation, a DT can allow prediction of ADs based on sparsely collected data (such as STP or pre-treatment imaging) without any additional scans after injection and manual dosimetry calculations (clinical workflow simplification). Therefore, although a DT itself is inherently complex, it can be regarded as a simplification approach consistent with the aim of the present study.

**Figure 5:**
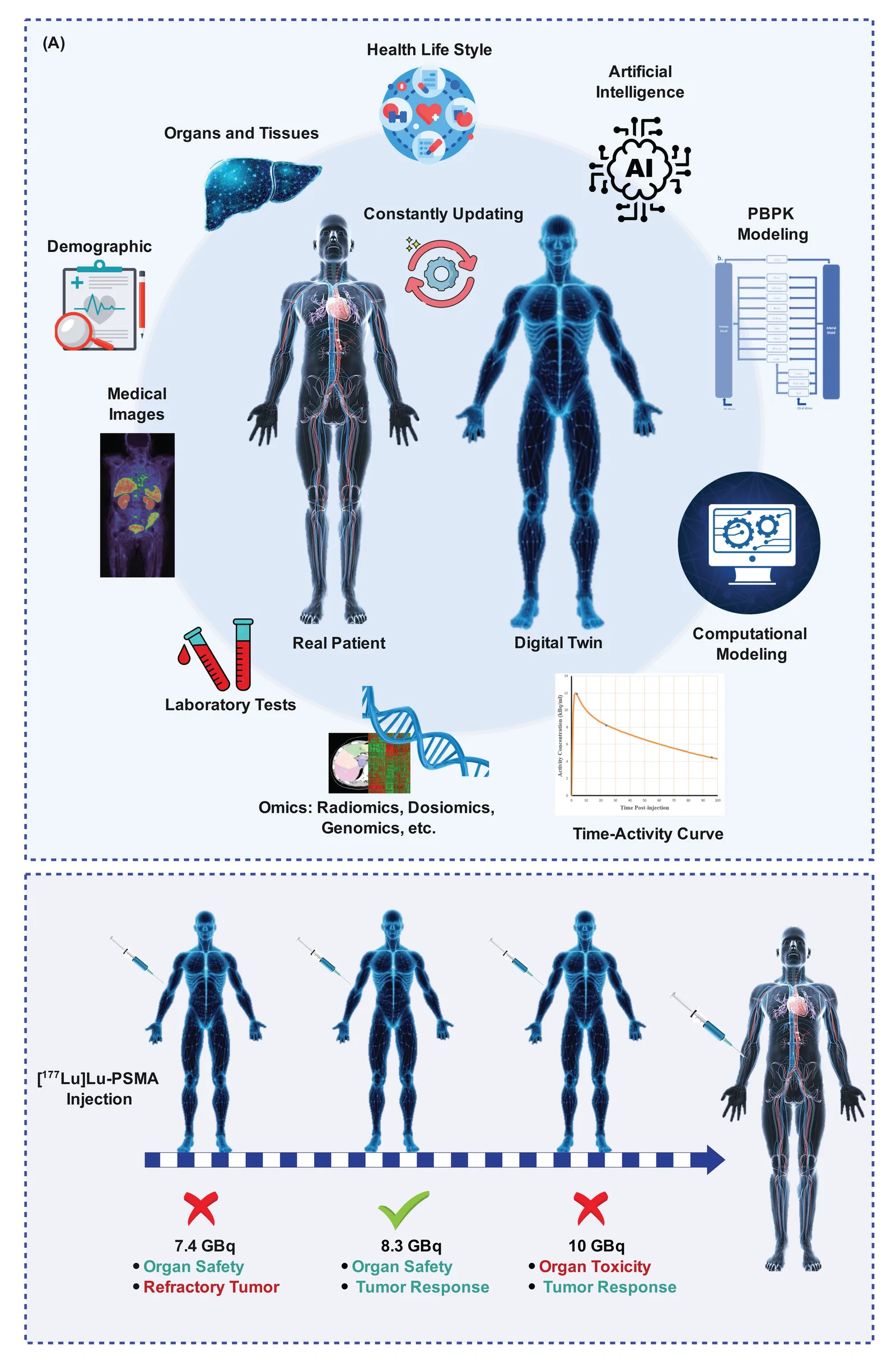
Theranostic digital twin for [^177^Lu]Lu-PSMA RLT. **(A)** Various modalities are combined to create personalized virtual patients. **(B)** Simulation reveals that 7.4 GBq is safe for organs and unsuccessful in tumor management, 8.3 GBq is successful in both, and 10 GBq causes toxicity; the clinical situation corresponded to 8.3 GBq.

Attempts to personalize RPT have resulted in the necessity to create theranostic DTs (TDTs), which consist of patient-specific, dynamically generated computational models, using data specific to patients, including clinical data, biomarkers, pharmacodynamic and pharmacokinetic data, and diagnostic images 12, 171, 172. These models simulate AD distribution and treatment outcomes, allowing personalized treatment planning and real-time modifications 173. Figure 5 describes the idea of personalized TDT and its use in designing [^177^Lu]Lu-PSMA RLT. Multimodal information from patients, such as demographic data, medical images, laboratory data, omics data, lifestyle factors, and TACs, is incorporated into computational models and PBPK models to create personalized virtual patients (Figure 5A). These DTs permit simulation of various administered activities to predict both response of the tumors and organs safety. For instance, based on the model (Figure 5B), administration of a fixed dose of 7.4 GBq ensures the safety of organs but results in a refractory tumor; a dose of 8.3 GBq ensures the safety of organs and the response of the tumor; a dose of 10 GBq ensures the response of the tumor, yet it causes organ toxicity. In the TDT-based real-world application of this case study, the injection is conducted at 8.3 GBq.

A four-step process towards the development of TDTs was introduced, including four major stages: (i) construction and parameter estimation of the model, (ii) optimization and customization of the model, (iii) calibration, improvement, and validation of the model, and (iv) implementation and ongoing modification of the model. This review emphasizes the benefits associated with customized treatment approaches for RPT along with their drawbacks. Improvements in this direction include data standardization, models customization, and model validation 12.

Mathematical and biokinetic models play an important role in establishing such schemes and are responsible for relating the inputs of treatment and their clinical consequences. Radiopharmaceutical pharmacokinetics deal with the uptake, distribution, and elimination of radionuclides in patients' bodies, whereas radiobiology describes the interaction of radiation and tissues 12. Such models rely on compartmental modeling, which means that the process of transferring radionuclides through the blood and body organs is represented by a system of differential equations. Compartmental differential equations allow the translation from physical AD to biological effects on molecular, cellular, and tissue levels. It becomes a basis of RPT with a biologically informed individual approach 12.

A population pharmacokinetic model was constructed by Siebinga *et al*. 174 consisting of six compartments to predict the pharmacokinetics and dosimetry of [^177^Lu]Lu-PSMA-617. This population pharmacokinetic model was successfully used with the help of imaging and blood samples of ten patients undergoing two cycles of treatment to describe both intra- and inter-patient variability, especially salivary gland uptake and tumor uptake. Saturability of salivary gland uptake and the effect of tumor volume on uptake rate are two important parameters of this model. Recent efforts involved the use of PBPK modeling along with DL for RPT 175.

Furthermore, PBPK modeling has been used to assess the impact of changes in infusion time and administration scheduling on ADs in tumors and OARs 176. Metronomic treatment schedules have been found to increase the ADs to the tumors and reduce their incidence on healthy tissues. In addition, the optimization of injection frequencies, intervals, and duration of infusion will result in more effective treatment without exceeding radiopharmaceutical boundaries.

An open-source computational model designed specifically for RPTs was proposed 99, 177. The method involved a scalable "reaction graph" paradigm enabling to integrate complicated biological processes. The created computational model considered the hot/cold ligand competition and differences in multiple bolus and single injection strategies, as well as different affinities to albumin.

However, converting such complex simulations into an actual workflow in clinical practice involves the use of computational techniques for integrating anatomical reality with the accuracy of AD computations. There are now computational mesh-based frameworks that allow accurate computation of the 3D AD at the organ level, small animal level, and cell-level modeling, without compromising compatibility with dosimetry data sources 178. Moreover, grid-based solutions for the linear Boltzmann transport equation (LBTE) also provide rapid voxel-based dosimetry with results comparable to MC simulations but with significantly reduced computation time, allowing its use in routine clinical applications 179. Various other methods of increasing efficiency, such as MC simulations using physics-driven patch cropping, proved that accurate dosimetry can now be done with much lower costs 166.

For such methods to be useful in clinical setting, complex mathematical models have to be modified and improved by simplifying them without losing any essential physiological information. This can be accomplished by applying techniques, such as the Manifold Boundary Approximation Method (MBAM), which simplify complicated models to allow for better usability and interpretability 180, 181. DTs combine the mechanisms of physiologically based modeling with the power of AI to provide accurate dosimetry for RPT.

While the above-mentioned simplification approaches can be considered separate areas to clarify understanding, they are not necessarily exclusive approaches to each other. Instead, they should be viewed as different methods addressing different steps in the dosimetry process workflow. Data acquisition is mainly minimized in STP techniques through the minimization of imaging time points. AI techniques aim to automate procedures like image correction, segmentation, registration, and AD computation. The PBPK model adds a mechanism through which radiopharmaceuticals behavior is described to make inferences about the pharmacokinetics of the drugs through sparsely sampled data. The DT model expands this approach through integration of individualized patient information with modeling and prediction.

**Figure 6:**
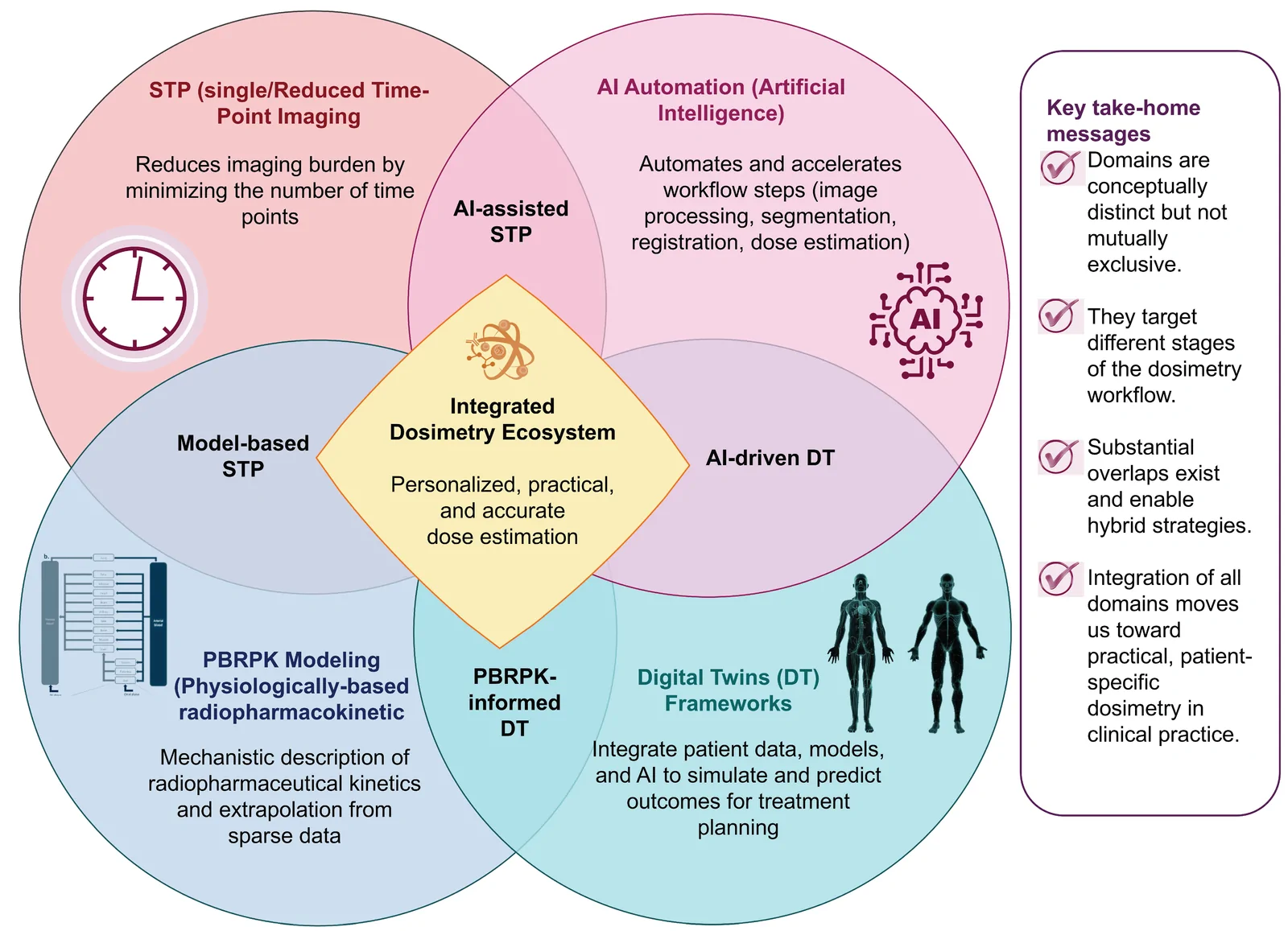
Conceptual depiction of the relationships among key dosimetry simplification domains. The single-time-point (STP) techniques mainly simplify imaging efforts; AI helps to automate the process; physiologically-based Pharmacokinetic (PBPK) models allow for kinetic modeling; while digital twins incorporate all of these into one model. The overlapped areas represent the use of hybrid concepts, such as AI-driven STP, PBPK-based estimation, and AI-driven digital twins (DTs).

Notably, the various domains also tend to overlap on many occasions. According to Figure 6, STP analysis could be coupled with PBPK modeling for more accurate kinetic analysis; AI technologies could complement STP-based AD assessment as well as PBPK modeling, while the concept of DT could involve both AI and PBPK techniques. It follows that these domains are not separate techniques but are different parts of one ecosystem of dose calculation approaches.

## 5. Discussion

Personalized dosimetry has become a vital part of the understanding of the changes that occur in lesions and OARs during RPT 182. However, the issue here is that current dosimetry methods are quite cumbersome, complicated, and demanding to perform, hence making their implementation into routine medical practice extremely difficult 19. It is necessary to simplify the dosimetry procedure without compromising its accuracy to render personalized dosimetry viable. In recent years, different solutions have been developed in different ways.

Until now, there is a lack of comprehensive reviews examining the different methods through which the dosimetry process could be simplified in relation to all RPTs and cancer cases. It is the objective of the present review to bridge the existing knowledge gap by discussing different innovations, such as STP imaging, automation through AI, and modeling using DTs, among others. The main challenge in dosimetry simplification is how to identify clinically relevant methods.

Moreover, there is lack of well-established evidence concerning the influence of simplification assumptions on the uncertainty of AD estimation. Regarding the issue under discussion, it should be mentioned that three notions need to be distinguished: "uncertainty" means total uncertainty about the estimation of AD and includes both systematic and random uncertainty; "accuracy" reflects how close the estimation using the simplified method is to the reference AD value and indicates the systematic uncertainty; finally, "precision" reflects the stability of the estimate in the case of repeated measurements in the same conditions and indicates random uncertainty. The definition of acceptable values of accuracy for simplified dosimetric techniques is extremely important for ensuring the appropriateness of clinical usage of such techniques, because the values of acceptable accuracy depend on different applications (tighter margins in the case of optimization of ADs of tumors and wider margins to control renal safety). Population-based techniques may reduce the number of imaging time points, but their efficiency was rarely investigated in a large population.

Propagated uncertainty is one of the challenges that is frequently overlooked in first-in-man dosimetry studies. According to the EANM guidance, sources of uncertainty are not limited to any one part of the work in question; rather, they arise from activity calibration measurements and counting statistics (both type A and type B), the selection of a proper mathematical model for fitting the TAC and extrapolation of kinetics in the late phase, organ delineation, and the choice of dosimetry modeling methods 67. It should be noted, as indicated in the EANM guidance, that a goodness-of-fit measure needs to be taken into account, along with the assessment of the effect of the sampling strategy, particularly the number of late-phase samples and its effect on TIACs 67. Otherwise, without the measure of uncertainty taken into account, estimates of AD values may seem to be more precise than they really are, taking into consideration the fact that pharmacokinetics are still largely unknown at the early phase 67. By using a log-normal scatter factor for propagation of the uncertainty, it becomes possible to calculate confidence intervals in accordance with the IAEA guidelines.

### 5.1. Clinically Ready Methods

In evaluating the closeness of different approaches to clinical practice, the following advances can be mentioned. Dosimetry with STP for stable organs, fewer time-point samplings, automatic segmentation and image registration, and MC simulation on GPUs have already shown the appropriate reliability for incorporation into clinical work. Quality assurance methods, such as polymer gel dosimetry, although not yet implemented, might prove extremely helpful for validating complex AD distributions, specifically in SIRT alone and in combination with EBRT 183, 184. In general, the above-mentioned methods are considered the most promising candidates for application in personalized dosimetry.

### 5.2. Emerging but Experimental Approaches

The application of AI for the reduction of time and increasing efficiency will provide another motivation for the inclusion of personalized dosimetry in routine procedures 9. AI can reduce time by reducing the requirement to conduct numerous SPECT scans and the number of time points needed to derive the TIAs. DL approaches for estimating and converting TIA into AD can serve as an inexpensive tool to avoid MC simulations without using generic anthropomorphic phantoms instead of patient's images 15. Moreover, AI-powered segmentation is under development for the improvement of individualization of image-guided RPT planning and follow-up procedures. The accurate determination of radiopharmaceutical distribution and the consequences thereof provide the means for evaluating the effectiveness of the treatment regimen and making adjustments. Finally, methods using patient data to optimize the imaging and dosimetry process will continue to increase in importance.

The application of pre-therapy patient data along with DT models appears to be a logical development in this area that allows a patient-focused approach, prediction of outcomes, and adjustment of AD based on specific needs. Although such approaches require further improvements to ensure replicability, reliability, and generalization, RPT dosimetry remains promising. Enhancing the spatial resolution of the imaging system would be a desirable method to reduce PVEs and enhance RPT dosimetric accuracy. Similarly, improving energy resolution would be desirable for reducing scatter, which would likely reduce the influence of scatter compensation on the uncertainty of AD estimates. The most recent significant advancement in SPECT camera hardware, which has been implemented in commercial systems, is the use of solid-state-based detectors. Cadmium-zinc-telluride (CZT)-based cameras offer better energy resolution and improved contrast by discriminating scatter, compared to Anger cameras that use scintillation crystals, such as NaI (Tl) 19.

With the enhanced applicability of multiparametric imaging along with predictive dosimetry through long axial field-of-view (LAFOV) PET/CT, there is potential of redefining the role of molecular imaging in the context of theranostics. LAFOV scanners provide great advancements in terms of increased sensitivity and improved coverage compared to conventional PET systems, thereby opening new avenues for using dual-tracer, delayed imaging, and dynamic imaging. It allows personalization of RPT by obtaining information required for the construction of PBPK models and TDTs 185.

As for future perspectives, the combination of AI and the use of PBPK models as a mechanistic approach, alongside DT model integration frameworks that consider multimodal data and simulation predictions, will likely redefine clinical practice in the next decade. The “AI-PBPK-DT” framework could allow for real-time AD prediction, cycle-by-cycle dose adjustment, and biological optimization of infusion procedures, multi-bolus dosing, and radioligand selection. However, this approach should be supported by proper data standardization and model validation 186.

### 5.3. Regulatory and Standardization Challenges

Of all the different simplification techniques, standardization proved to be the most pressing issue. Indeed, several aspects of the problem appeared, including: (i) acquisition protocols, such as scanning time points, scanner calibration and reconstruction parameters; (ii) data analysis procedures, such as segmentation and registration algorithms; (iii) model construction and validation methods, such as reference standards, parameter optimization and validation schemes; and (iv) evaluation methods, i.e. performance metrics, uncertainty estimation and repeatability measures. These interconnected issues need to be solved to enable a reliable comparison of different simplified dosimetry approaches.

Data obtained from the SNMMI ^177^Lu Dosimetry Challenge revealed that, even for experienced centers, significant differences still exist in the estimation of AD via segmentations, fittings, or conversions of TIACs to AD techniques 61, 187-189. This is a clear indication that accuracy cannot be assured simply through simplification. Validation, assessment, and clinical acceptance of any simplification method is entirely based on comparison to a well-defined set of standard data to ensure that what is being called accurate is not simply a system bias. For reproducibility in dosimetry, harmonization of image acquisition processes, processing steps, and assessment criteria is needed.

A recent meeting held by more than 180 experts from the European Federation of Organizations for Medical Physics (EFOMP) noted increasing interest and willingness of medical physicists to promote patient-specific dosimetry 190. Efforts are being made to make dosimetry more accurate, reliable, and traceable, with AI likely making contributions towards automation and standardization. The introduction of dosimetry into medical practice prior to the results of trials ensures better monitoring and decision-making regarding repeat treatments, thus providing benefit to patients in the long run.

However, for dosimetry to become successful, the need exists to introduce it in the early stages of development of radiopharmaceuticals to end the vicious circle of insufficient evidence and delayed implementation of dosimetry into practice. In parallel, discussions are taking place amongst radiation oncologists and hematologists on the need for adaptive or personalized dosing versus conventional dosing of patients with novel therapies 191. Additionally, the EANM is supporting the implementation of personalized dosimetry 5.

Although AI shows great promise in the field of theranostics, there are still ethical concerns regarding the issues of patient privacy, confidentiality of information, and ownership. Federated learning may help resolving these problems, as it allows training ML and DL models with large and diverse datasets that do not require transferring or sharing the data locally. This type of machine learning is key to ensuring that AI remains sustainable in theranostics through sufficient training and validation with a sufficiently large and diverse dataset, thus increasing its external validity and generalization. In this review, we discuss the latest developments in AI that can help in performing RPT dosimetry in a more precise manner, taking advantage of the unique possibility to personalize the process of RPT dosimetry.

Another benefit that arises from the use of AI in clinical dosimetry is the development of explainable AI (XAI), whereby AI offers transparency in relation to its decision-making process. Through the use of XAI, clinicians can get explanations regarding how AI makes predictions concerning AD or RPT outcomes, thus promoting trust and sound decision-making 192-194. The combination of XAI and the DT approach could increase confidence and transparency in personalized RPT planning.

The field of implementation science (IS) recognizes that effective implementation of AI in medical imaging cannot rely only on strong evidence-based research and technical advances but must also consider other aspects of human interaction, cooperation, and joint ownership. According to the principles of IS, true impact on real-world healthcare delivery and outcomes can only be achieved through intersectoral collaboration involving many parties 195. The same logic applies to personalized dosimetry workflows of internal radiation exposure in RPT. Implementation of AI-based tools for automated segmentation, AD calculation, and workflow optimization will never result in clinical success without close collaboration between many players, including nuclear medicine physicians, medical physicists, technologists, software specialists, and IT personnel. It is also important to remember that implementation of innovations should involve joint ownership and open dialogue between the parties involved to ensure that clinical needs are understood and addressed. Finally, due to the multi-institutional nature of internal dosimetry, the development of standards, data sharing strategies, and multi-center validation projects will be key elements of successful implementation, just like in implementation science.

The clinical deployment of AI-powered dosimetry applications, PBPK models, and DT systems would need proper regulatory positioning as well as validation of the technologies used. The main differentiation here will be between clinical decision support (CDS) solutions that aid clinicians but can be independently verified and the software as a medical device (SaMD) application that informs or automates clinical decisions and thus should be regulated more strictly. The application of AI for AD estimation and DT systems is likely to pose high risk due to their potential impact on treatment decisions.

Implementation in clinical setting requires showing that there is analytical validity, where it is demonstrated that the technique is equivalent to the results of MC dosimetry, clinical validity, and reliability in various patients. In this sense, one of the key issues to overcome is the limited generalizability associated with the training of many models using a dataset from only one research center. The differences in scanners and image analysis software can seriously affect the results. In terms of PBPK and DT modeling, other issues to consider are the need to validate these techniques in prospective studies and make the interpretation of such models as transparent as possible. Despite the growing recognition of these methods in regulatory science, their clinical use will be contingent upon their reproducibility and clinical effectiveness.

### 5.4. Research Priorities

One of the limitations in the implementation of personalized RPT is the absence of AD models for radiobiology. Radiobiology is crucial in gaining a mechanistic understanding of the efficacy and toxicities of the agents. Nevertheless, the field of radiobiology has traditionally been concerned with the use of EBRT. There are important differences between RPT and EBRT concerning characteristics, such as the type of dosimetry, the dose rate, LET, time of administration of the treatment, fractionation, range, and target volume. In the case of RPT, ADs are several orders of magnitude smaller, have large heterogeneity in space and time, and contain microdosimetric effects for α-particle-emitting agents 196-198. This makes it challenging to translate the findings on the radiobiology of EBRT into RPT and also present a methodological caveat in the study of RPT. The above problem can be solved by the adoption of low-AD-rate biologically effective doses (BED), particularly by adopting corrections for AD-rate, such as the Lea-Catcheside factor *G*, which corrects for the rate of DNA repair relative to drug clearance half-life 101, 199.

For [^177^Lu]Lu-PSMA-617, for example, a dosimetry study currently in progress has indicated that radiobiologically correcting the BED or EQD2 in 2-Gy AD can substantially alter the calculated therapeutic index. For instance, if high AD is administered to the tumor, the tumor-to-kidney dose ratio may even reduce by as much as 30% compared with only AD 197. This indicates that the safety limits derived from EBRT can grossly underestimate safety limits in RPT 200. For the calculation of BED and subsequent adaptive dosing, individual clearance half-lives of each radiopharmaceutical must be taken into account. This will help to lower the level of activity in those subjects who demonstrate slower rates of clearance.

In addition to the modeling of BED, there is an acute need for further radiobiological studies for RPT. These studies ought to consider repair mechanisms, dose-effect relations on the scale of individual organs and voxels, and microdose evaluation of alpha-radiopharmaceuticals 201. Nonetheless, for these findings to make a difference in the clinic, they need to be transformed into applicable concepts, such as clearance-based dose restrictions, carefully planned multicenter clinical trials, and incorporation into treatment planning systems. Without such a step of translation, RPT is likely to remain merely a physical procedure, not a biological one.

### 5.5. Health-Economic Considerations

A brief overview of the cost-effectiveness of simplified dosimetry is necessary to comprehend the implications of simplified dosimetry in a policymaking setting. On one hand, AD-guided treatment regimens incur costs due to increased imaging requirements, software usage, and personnel costs; on the other hand, fixed-activity administration incurs costs in the form of suboptimal response. Fixed-activity protocols may result in undertreatment (wasted drug, additional cycles, disease progression) or overtreatment (toxicity management, hospitalization). AD-guided treatment protocols require extra costs per patient but offer certain offset opportunities: reduction of non-responders prevents unnecessary drug costs; prompt detection of fast clearance allows for increasing AD without extra toxicity; prevention of extreme toxicity lowers hospital admissions and supportive care expenses; and STP/automation considerably cuts down costs associated with dosimetry when compared to MTP-based dosimetry. Some indications suggest that simplified dosimetry is cost-effective in high-volume centers, but there is lack of data from health economic studies comparing simplified dosimetry vs. fixed activity administration across diverse healthcare settings. Lack of proper reimbursement is another important obstacle, since dosimetry is frequently not paid for or needs to be justified to a considerable extent.

### 5.6. Summary

In essence, the move towards clinical practice through personalized dosimetry must be accompanied by an approach focusing not only on feasibility but also on scientific evaluation. Multicenter prospective studies and unified imaging protocols defined uncertainty calculations, as well as randomized clinical trials comparing the outcome of personalized dose calculations based on anatomic dosimetry compared with a fixed activity protocol represent the cornerstones. Likewise, health economics analyses may have a significant impact on the decision-making process for clinical adoption, as financial burden represents another important hurdle faced by most centers.

As broad as it is, this review has a few limitations as well. First of all, studies on the topic of dosimetry simplification differ considerably in terms of image acquisition, image reconstruction procedures, pharmacokinetics hypotheses, segmentation techniques, and evaluation measures, thus hindering comparisons. Secondly, some areas, such as voxel dosimetry based on AI or DT ecosystems might be understudied because of their rapid development and recent publication date. Thirdly, almost no method is externally validated, as studies are mostly single-center and retrospective. Lastly, although the main focus was on synthesis and comparison of concepts, specificities related to certain radiopharmaceuticals and/or tumor sites may be underestimated.

Combined, these pieces of evidence point to a conclusion that simplification of dosimetry is by no means a shortcut; rather, it is a calculated move that permits a reliable application of RPT on an individual basis. Through continued research and innovation as well as tight cooperation between the parties involved in the development and validation process, simplified dosimetry can personalize RPT in the future.

## 6. Conclusions

RPT dosimetry simplification is no longer a set of independent measures but rather an integrated process. Various techniques, including STP imaging, AI-powered automation, PBPK models, and DT frameworks, tackle specific problems within the whole chain of actions. Each area can reduce complexity separately, but in combination, they offer the largest possibilities for clinical practice innovation. Reduced imaging procedures in tandem with the use of AI and PBPK models allow estimating accurate AD for a patient without requiring excessive resources. A paradigm of DT frameworks incorporating various sources of patient information in one complex tools seems very promising. From a medical perspective, this means that the transformation of dosimetry from a time-consuming research tool into a routine process of healthcare delivery with the possibility of personalizing the treatment of patients, improving its outcomes, and increasing safety. Yet, there are still many problems that need to be addressed. Realizing the full potential of RPT requires the development of integrated, effective, and automated dosimetry techniques. AI, mechanistic modeling, and DTs will have a pivotal role in achieving this goal.

## Figures and Tables

**Figure 3:**
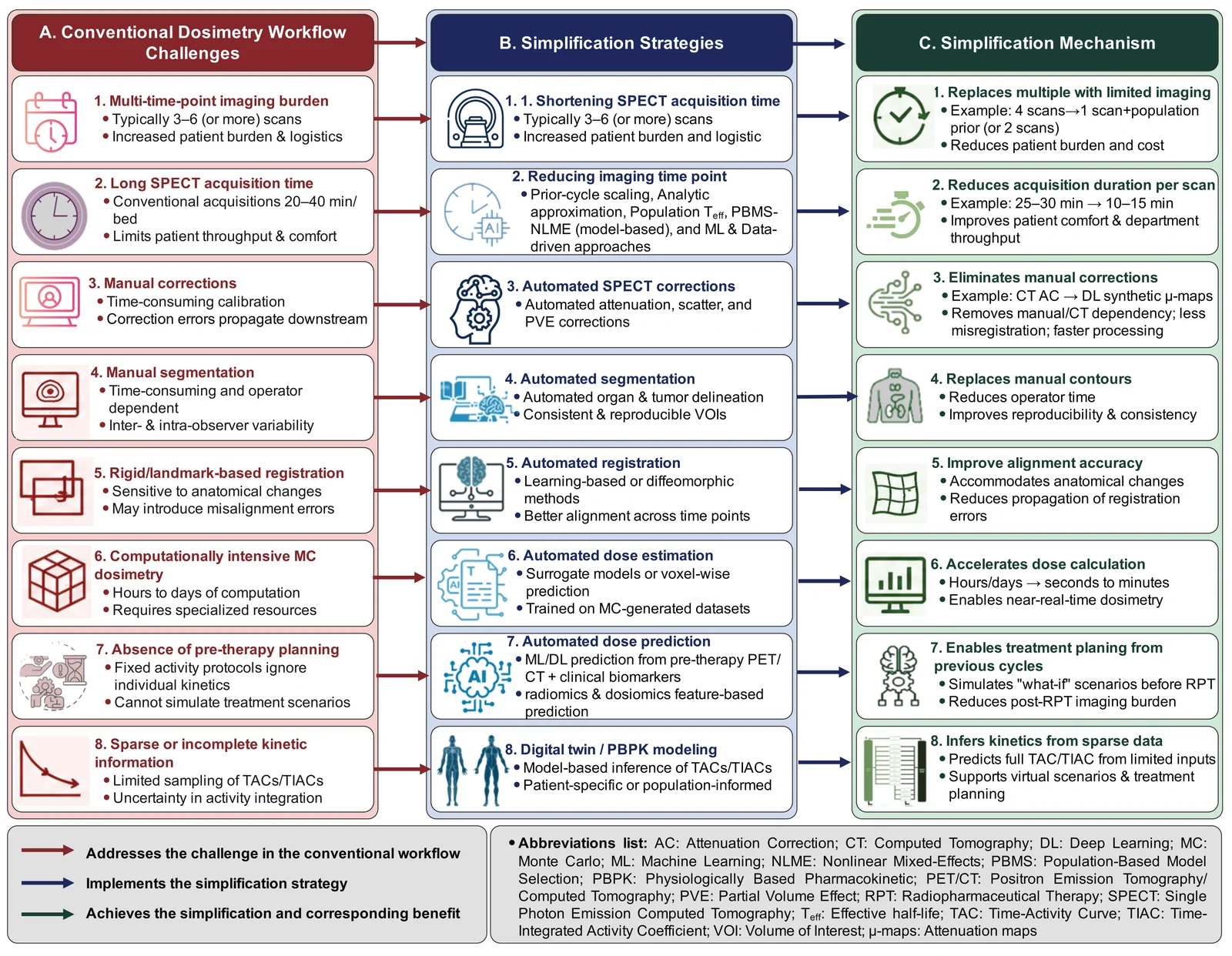
Diagrammatic representation of the mapping of dosimetry workflow challenges, possible simplification strategies for overcoming these problems, and the subsequent simplification mechanism. The left column provides some important problems in the dosimetry workflow of standard radiopharmaceuticals. The middle column describes the simplification techniques that can be employed to overcome these problems. The right column explains how simplification is achieved and the gain from each technique.

**Table 1:** Overview of key therapeutic radiopharmaceuticals, their clinical applications, and related dosimetric challenges.

Therapy / Application	Radionuclide (Emission)	Target / Indication	Regulatory Status	Key Dosimetric Challenge	Ref.
Radioiodine Therapy	[¹³¹I]NaI (β⁻, γ)	Differentiated thyroid cancer	FDA/EMA approved	Variable iodine uptake; nonlinear biokinetics	29, 30
Bone-Targeted Therapy (palliation)	[⁸⁹Sr]SrCl₂ (β⁻, γ), [¹⁵³Sm]Sm-EDTMP (β⁻, γ)	Bone metastases (BCa, PCa); pain palliation	FDA/EMA approved	Heterogeneous uptake in bone microenvironment; limited γ emissions complicate dosimetry and imaging	31-33
Bone-Targeted α-Emitter	[²²³Ra]RaCl₂ (α, γ)	Bone metastases (mCRPC, BCa)	FDA/EMA approved	Microdosimetry at bone–marrow interface; low γ yield for imaging	34
Liver Radioembolization (SIRT/TARE)	[⁹⁰Y]Y-microspheres (β⁻)	HCC, liver-dominant metastases	FDA/EMA approved	Nonuniform microsphere distribution; heterogeneous absorbed dose deposition in tumor vs. normal liver	35
Liver Radioembolization (Holmium-based)	[¹⁶⁶Ho]Ho-PLL-microspheres (β⁻, γ)	Liver metastases, HCC	Clinically available in some regions (EU)	Nonuniform distribution; potential for MRI-based dosimetry via paramagnetic properties	36, 37
PRRT (DOTATATE / DOTATOC)	[¹⁷⁷Lu]Lu-DOTATATE/ DOTOTOC (β⁻, γ), [⁹⁰Y]Y-DOTATATE/ DOTOTOC (β⁻, γ)	NETs	FDA/EMA approved	Inter-cycle variability: kidney and bone marrow absorbed dose constraints	38, 39
mIBG Therapy	[¹³¹I]I-mIBG (β⁻, γ)	Neuroblastoma, pheochromocytoma, sympathetic tumors	FDA/EMA approved	Nonuniform tumor uptake, OAR overlap (liver, marrow)	40
PSMA RLT (mCRPC)	[¹⁷⁷Lu]Lu-PSMA-617 (β⁻, γ)	mCRPC, Targets PSMA-expressing cancer cells	FDA/EMA approved	Heterogeneous PSMA expression; lack of α-imaging for α-emitter versions	41-43
PSMA RLT (α-Emitter)	[²²⁵Ac]Ac-PSMA (α, γ)	mCRPC	Investigational (Phase I/II)	Microdosimetry; daughter radionuclide redistribution; kidney/marrow toxicity	44
SSTR2-Targeted α-Therapy (Antagonist)	[^225^Ac]Ac-SS0110	SSTR2+ cancers (e.g., SCLC, MCC)	Investigational (Phase I/II)	Microdosimetry; daughter radionuclide redistribution; kidney/marrow dosimetry; heterogeneous uptake	24
Albumin-binding α-Emitter	[²²⁵Ac]Ac-ABD-147 (α, γ)	Lung and NETs	Investigational (Phase I/II)	Prolonged circulation increases OAR absorbed dose; need for patient-specific modeling	45
α-Emitter Prodrug Targeting Melanoma	[²¹²Pb]Pb-VMT01 (α, γ)	Metastatic melanoma	Investigational (Phase I/II)	Rapid progeny recoil: dosimetric modeling of daughter radionuclide distribution	46
Auger/ Mixed Emitters	[¹⁶¹Tb]Tb (β⁻, Auger electron)	Micrometastatic disease (e.g., mCRPC)	Investigational (Phase I/II)	Subcellular-level energy deposition; need for accurate intracellular localization	47
Intra-tumoral RLT	[¹⁸⁶Re]Re-RNL (β⁻, γ)	Gliomas, liver tumors, bone metastases	Limited approvals/ Investigational	Local distribution challenges; complex intratumoral dosimetry	48

**Abbreviations list:** BCa: Breast Cancer; HCC: Hepatocellular Carcinoma; HNC: Head and Neck Cancer; mCRPC: Metastatic Castration-Resistant Prostate Cancer; MCC: Merkel Cell Carcinoma; OAR: Organs at Risk; PCa: Prostate Cancer; PRRT: Peptide Receptor Radionuclide Therapy; PSMA: Prostate-Specific Membrane Antigen; Ref: reference; RLT: Radioligand Therapy; SIRT: Selective Internal Radiation Therapy; SCLC: Small Cell Lung Cancer; SSTR2: Somatostatin Receptor Type 2; TARE: Transarterial Radioembolization; mIBG: Meta-Iodobenzylguanidine; NET: Neuroendocrine Tumor.

**Table 2:** Conventional dosimetry workflow: limitations and proposed simplifications.

Standard Step	Key Limitation(s)	Proposed Simplification (Section)
3.1 Data acquisition	MTP imaging (3-4 scans over 4-7 days): high cost, patient burden, clinic logistics, scanner availability; the need for attenuation, scatter, and partial volume corrections	Reduced-time-point / STP imaging (4.2); shortened SPECT acquisition (4.1); DL-based corrections (4.3)
3.2 Registration & segmentation	Manual delineation: time-consuming, operator-dependent, inter-/intra-rater variability	Automated segmentation (4.4); automated registration (4.5)
3.3 Dose conversion modeling	Organ-level S-values: ignore patient anatomy and heterogeneity; voxel-based methods: computationally intensive	DL-assisted absorbed dose estimation (4.6); hybrid DL-MC methods (4.6)
3.4 Integration (TIA/TIAC)	Requires MTP data; curve fitting is sensitive to TP selection, and extrapolation	STP methods (4.2); PBPK/NLME models (4.2.4, 4.8); data-driven GAM/ML (4.2.5)
3.5 Absorbed dose calculation	MC simulations: hours–days; specialized expertise; not routine	DL absorbed dose estimation (4.6); absorbed dose prediction (4.7), kernel methods with DL refinement (4.6)
Across all steps	Manual interventions, error propagation, and lack of standardization	Digital twin frameworks (4.8); integrated AI pipelines (4.3–4.6)

**Abbreviations list:** DL = deep learning; GAM = generalized additive model; MC = Monte Carlo; ML = machine learning; MTP = multi-time-point; NLME = nonlinear mixed-effects; PBPK = physiologically based pharmacokinetic; STP = single-time-point; TIA = time-integrated activity; TIAC = time-integrated activity coefficient; TP = time point; VSV = voxel S-value.

**Table 3:** Simplified approaches for dosimetry: standard of reference, clinical challenge, validity conditions, and conclusion.

Technique	Standard of Reference	Clinical Challenge	Validity Conditions	Conclusion
1. Reducing SPECT acquisition time	Full-duration SPECT (25-30 min per bed)	Extended scan time, patient discomfort, and reduced throughput	Count statistics are adequate; DL models are validated locally	Reduce scan time by 50-75% with DL; validate before clinical use
2. 1. Reducing imaging time point: Prior-cycle scaling	First-cycle MTP (3-4 time points)	Tumor kinetics change across cycles; response may alter uptake	Stable organ kinetics; validated for a specific radiopharmaceutical	Acceptable for renal safety monitoring; not for tumor absorbed dose tracking
2.2. Reducing imaging time points: Analytic approximation (Hänscheid)	Full MTP (3-4 time points over 4-7 days)	TP selection critical; mono-exp assumption may be mis-specified	TP at 72-96 h; mono-exponential kinetics are appropriate	Simple but TP-dependent; sometimes two time points preferred over one
2.3. Reducing imaging time points: Population T_eff_~ (Madsen)	Full MTP (3-4 time points over 4-7 days)	Inter-patient variability; outliers produce large errors	Well-characterized population half-life; organs with predictable kinetics	Practical for routine organ dosimetry and lesions with predictable kinetics, but TP-dependent and sensitive to outliers.
2.4. Reducing imaging time points: PBPK and NLME modeling approaches	ATP with AICc/Akaike-selected model; large population cohort	Requires population cohort; NLME expertise; computational resources	Large population priors; AICc-based model selection; regulator-recognized	Most defensible benchmark; regulator-recognized; preferred for accuracy
2.5. Reducing imaging time points: Machine Learning and Data-Driven Approaches	Full MTP (3-4 time points over 4-7 days) with traditional analytical methods (Madsen, Hänscheid)	Traditional methods assume fixed functional forms; fail to capture interpatient variability; and cannot incorporate patient-specific biomarkers	Large training datasets; leave-one-out cross-validation; validated for specific radiopharmaceuticals and time points; biomarkers (e.g., eGFR, tumor volume) available	Might outperform traditional methods; integrating biomarkers enhances accuracy; might be promising for early time points and atypical kinetics, data hunger
3. Automated SPECT corrections	Manual correction (CT-AC, DEW or TEW scatter, RC)	Operator time; inter-operator variability; misregistration	DL models trained on representative data; large, diverse, and multi-center validation	Reduces operator time; validates generalizability
4. Automated segmentation	Manual delineation by an expert	Time-consuming inter- and intra-observer variability	High-contrast organs/tumors; DL model validated on similar data	Reliable for organs; requires caution for lesions
5. Automated registration	Manual/rigid registration of serial scans	Motion misalignment; deformable anatomy; operator dependence	SPECT-CT alignment; deformable motion in abdomen/thorax and H&N	Deformable DL registration reduces operator time and improves accuracy
6. Automated absorbed dose estimation	Monte Carlo simulation	Computational time (hours to days); specialized expertise/	Homogeneous tissues acceptable; DL-MC hybrid for heterogeneity	DL enables real-time absorbed dose estimation; MC remains the gold standard
7. Absorbed dose prediction (pre-therapy)	MTP post-therapy dosimetry	Cannot personalize first cycle; delayed treatment adaptation	Stable disease; similar tracer kinetics; validated models	Useful for cycle 1 planning; not yet validated for all therapies
8. Digital twin / PBPK modeling	MTP imaging + MC dosimetry	Model complexity; parameter uncertainty; validation needed	Sufficient training data; prospective validation	Experimental; not yet ready for routine clinical use

**Abbreviations list:** AC = attenuation correction; AICc = corrected Akaike Information Criterion; ATP = all-time-point; DEW = dual-energy window; DL = deep learning; DSC = Dice similarity coefficient; MAE = mean absolute error; MC = Monte Carlo; MTP = multi-time-point; PBMS = population-based model selection; PBPK = physiologically based pharmacokinetic; PVC = partial volume correction; RC = recovery coefficient; RMSE = root mean square error; SC = scatter correction; STP = single-time-point; TEW = triple-energy window; TP = time point.

**Table 4:** Conventional and deep learning-based methods for SPECT imaging data correction.

Correction Method	Conventional Methods	DL-based Methods	Advantages of DL-based Correction	Limitations/Challenges
Attenuation correction	CT-based μ-maps generation, segmentation, and MLAA	Surrogate μ-maps or AC maps obtained from non-AC images 106, 109, 112, 113	Insensitive to misregistration errors; less complicated process; potential for CT-free correction	Requirement of a large number of training data; low generalizability capability
Scatter correction	TEW model; convolution-kernel method; MC	Scatter estimation using CNN and transformers; combined AC and SC models 109, 114-116	Fast algorithm; high accuracy comparable with MC; enhanced lesion quantification; reduced manual intervention	Variation depending on the radionuclide and the scanner; need for multi-center study
PVE / resolution correction	Recovery coefficients and deconvolution with segmentation	DL-PVC from MC simulations or phantoms 105, 110, 117, 118	No need for segmentation; noise resistance; enhanced quantitative accuracy and artifact removal	Susceptible to misregistration between SPECT and CT scans; inadequate investigation for organ motion
Other Relevant Corrections	Analytic denoising; conventional positron-range modeling	Diffusion-based denoising; positron-range correction via DL 119-121	SNR improvement with details retained; quantification accuracy in PET/SPECT imaging	Over-smoothing problem; restricted evaluation of therapeutic isotopes

**Abbreviations list:** AC: attenuation correction; CNN: convolutional neural network; DL: deep learning; MC: Monte Carlo; MLAA: maximum-likelihood attenuation and activity; PVC: partial volume correction; SC: scatter correction; SNR: signal-to-noise ratio; TEW: triple-energy window.

## Abbreviations

3D: Three-Dimensional
AC: attenuation correction
AD: Absorbed Dose
AI: Artificial Intelligence
ATP: All-Time-Point
BED: Biologically Effective Dose
BM: Bone Marrow
BREP: Boundary Representation
CB: Clinical Biomarker
CDS: clinical decision support
cGAN: Conditional Generative Adversarial Network
CNN: Convolutional Neural Network
CT: Computed Tomography
DL: Deep Learning
DNN: deep neural networks
DSC: Dice Similarity Coefficient
DT: Digital Twin
DVK: Dose Voxel Kernel
DVH: Dose–Volume Histogram
DTC: Differentiated Thyroid Carcinoma
EBRT: External Beam Radiotherapy
eGFR: estimated Glomerular Filtration Rate
EMD: empirical mode decomposition
EQD2: equi-effective dose in 2 gray fractions
FM: Fixed Method
FS: Fourier Surface
GAN: Generative Adversarial Network
GPU: Graphics Processing Units
HU: Hounsfield Unit
iSTP: instant Single-Time-Point
ITM: Image Thresholding Method
LBTE: Linear Boltzmann Transport Equation
MAA: Macroaggregated Albumin
MAE: Mean Absolute Errors
MAPE: Mean Absorbed Percentage Error
MC: Monte Carlo
mCRPC: Metastatic Castration-Resistant Prostate Cancer
MIRD: Medical Internal Radiation Dose
ML: Machine Learning
MPA: maximum permissible activity
MRAE: Mean Relative Absolute Error
MRI: Magnetic Resonance Imaging
MRT: Molecular Radiation Therapy
MS: Model Selection
MSV: Multiple S-Value
MTP: Multiple Time-Point
NET: Neuroendocrine Tumor
NLME: Nonlinear Mixed-Effects
NMAE: normalized mean absolute error
NRMSE: Normalized Root-Mean-Square Error
OAR: Organ-at-Risk
OM: Otsu Method
OS: Overall Survival
OSEM: Ordered Subset Expectation Maximization
PBPK: Physiologically Based pharmacokinetic
PBMS: Population-Based Model Selection
PET: Positron Emission Tomography
PFS: Progression-Free Survival
PRRT: Peptide Receptor Radionuclide Therapy
PSMA: Prostate-Specific Membrane Antigen
PVE: Partial-Volume Effects
RAIT: Radioactive Iodine Therapy
RIThM: Recovery Iterative Thresholding Method
RMSE: Root Mean Square Error
RPT: Radiopharmaceutical Therapy
SAM: Segment Anything Model
SaMD: software as a medical device
SBRT: Stereotactic Body Radiation Therapy
SC: scatter correction
SIRT: Selective Intra-Arterial Radiation Therapy
SM: Simplified Method
SNMMI: Society of Nuclear Medicine and Molecular Imaging
SSIM: Structural Similarity Index Measure
SSL: Self-Supervised Learning
SSV: Single S-Value
SUV: Standardized Uptake Value
SPECT: Single Photon Emission Computed Tomography
SSTR: Somatostatin Receptor
STP: Single Time Point
Swin UNETR: Shifted Windows UNEt Transformers
TAC: Time–Activity Curve
TARE: transarterial radioembolization
TDT: Theranostic Digital Twins
TIA: Time-Integrated Activity
TIAC: Time-Integrated Activity Coefficient
TTP: Two-Time-Point
VAA: Volume Activity Accuracy
VSV: Voxel S-Value
VOI: Volume of Interest
wDice: Weighted Dice
XAI: Explainable AI
